# Blockage of Squamous Cancer Cell Collective Invasion by FAK Inhibition Is Released by CAFs and MMP-2

**DOI:** 10.3390/cancers12123708

**Published:** 2020-12-10

**Authors:** Inés Sáenz-de-Santa-María, Lucía Celada, Andrés San José Martínez, Tamara Cubiella, María-Dolores Chiara

**Affiliations:** 1 Instituto de Investigación Sanitaria del Principado de Asturias, 33011 Oviedo, Spain; ines.ssantamaria@gmail.com (I.S.-d.-S.-M.); celadalucia@hotmail.com (L.C.); andres.sanjosem@gmail.com (A.S.J.M.); tamaracubiella@gmail.com (T.C.); 2 Unit of Membrane Traffic and Pathogenesis, Institute Pasteur, 75015 Paris, France; 3 CIBERONC, 28029 Madrid, Spain; 4 Instituto Universitario de Oncología del Principado de Asturias, Universidad de Oviedo, 33006 Oviedo, Spain

**Keywords:** metastasis, collective migration, focal adhesion kinase, matrix metallopeptidase 2, cancer-associated fibroblasts, squamous cell carcinomas

## Abstract

**Simple Summary:**

Cancers include a diverse collection of cells harboring distinct molecular signatures with different levels of pro-metastatic activities. This intratumoral heterogeneity and phenotypic plasticity are major causes of targeted therapeutic failure and it should be considered when developing prognostic tests. Through the analysis of the Focal Adhesion Kinase (FAK) protein and the matrix metalloprotease MMP-2, both implicated in multiple steps of the metastatic spectrum, in complex multicellular tumor spheroids we show that cancer cell populations over-expressing MMP-2 or cancer-associated fibroblasts can release FAK-deficient cancer cells from their constrained metastatic fitness. Consistently, MMP-2, not FAK, serves as an independent prognostic factor in head and neck squamous cell carcinomas. Measurement of intratumor heterogeneity facilitate the development of more efficient biomarkers to predict the risk of metastasis and of more-effective personalized cancer therapies.

**Abstract:**

Metastasis remains a clinically unsolved issue in cancer that is initiated by the acquisition of collective migratory properties of cancer cells. Phenotypic and functional heterogeneity that arise among cancer cells within the same tumor increase cellular plasticity and promote metastasis, however, their impact on collective cell migration is incompletely understood. Here, we show that in vitro collective cancer cell migration depends on FAK and MMP-2 and on the presence of cancer-associated fibroblasts (CAFs). The absence of functional FAK rendered cancer cells incapable of invading the surrounding stroma. However, CAFs and cancer cells over-expressing MMP-2 released FAK-deficient cells from this constraint by taking the leader positions in the invasive tracks, pushing FAK-deficient squamous cell carcinoma (SCC) cells towards the stroma and leading to the transformation of non-invasive cells into invasive cells. Our cell-based studies and the RNAseq data from the TCGA cohort of patients with head and neck squamous cell carcinomas reveal that, although both FAK and MMP-2 over-expression are associated with epithelial–mesenchymal transition, it is only MMP-2, not FAK, that functions as an independent prognostic factor. Given the significant role of MMP-2 in cancer dissemination, targeting of this molecule, better than FAK, presents a more promising opportunity to block metastasis.

## 1. Introduction

Cancer metastasis is the leading cause of cancer-related death and, thus, unraveling the mechanisms of this complex process is an effective approach to increasing our ability to effectively target and treat this disease to decrease patient morbidity and mortality. The metastatic fitness of cancer cells is achieved by the acquisition of migratory capacity, alteration of the adhesion of cells to the extracellular matrix (ECM), and remodeling and proteolysis of this matrix. Two distinct patterns of tumor cell migration and invasion have been described thus far: single-cell migration and collective migration of multi-cellular clusters [1,2]. Both types of migration rely on the polarized cell–ECM interaction and cell-generated mechanical forces that, in the case of collective migration, mainly occur at the free margin of the cellular clusters [3]. It has become apparent that multi-cellular cluster migration is usually led by a subset of “leader and invasion-competent cells” that show the highest capacity for proteolytic ECM remodeling and induce the collective invasion of otherwise “followers and invasion less-competent” epithelial cells. These interactions are inherently very complex during the collective migration of mechanically coupled cells because of the additional regulation of cell–cell junctional forces transmitted across cadherin adhesions [4,5]. Integrins provide polarized interactions with the ECM ligands at the protruding extensions of invasion-competent cells which act as leaders of the migration tracks [6,7,8]. Once integrins interact with ECM, focal adhesion complexes assemble by the recruitment and activation of cytoplasmic signaling proteins, including Focal Adhesion Kinase (FAK) [9]. Then, leader cells initiate protease-driven tissue remodeling, creating open spaces for the advancing cell mass. It has become evident that leader cells are the ones that have the highest capacity for proteolytic ECM remodeling via MMP-2 upregulation and redistribution [10].

FAK acts as a critical bidirectional linkage between the actin cytoskeleton and the cell–ECM interface that mediates signaling events that facilitate single-cell migration and invasion through the regulation of the activity of the Rho family GTPases [11] and the up-regulation of several proteases, such as the metalloprotease MMP-2 [12,13]. Several lines of evidence have shown that integrins and FAK become integrated and coordinated with cell–cell adhesion receptors although mechanisms and crosstalks during collective cell migration are not yet fully understood. For instance, FAK has been shown to be required for the assembly of the cadherin adhesion complex that guides collective migration during embryonic development [14] while other studies have shown that FAK controls the epithelial to mesenchymal transition (EMT) program, such that embryonic FAK-null cells are committed to an epithelial status highlighted by the expression of E-cadherin and cytokeratins [15]. There are also evidences for crosstalks between FAK and E-cadherin in cancer but, again, the data are limited and conflicted. Decreasing FAK activity has been shown to maintain cell–cell adhesion and turn dispersed cells that migrate as single-cells into multicellular clusters that migrate collectively, while increased FAK pathway activity de-regulates E-cadherin and drives EMT, which disrupts cell collectives and promotes individual cell migration and a more motile phenotype [16,17,18]. Other studies, however, showed that inhibition of FAK activity abrogates collective movement yet induces the detachment of individual amoeboid cells from the collective or that FAK-mediated signaling has a positive effect on cell–cell contact formation via induction of E-cadherin [19,20,21,22,23]. These findings illustrate that FAK’s role in regulating intercellular adhesion and collective migration is extremely complex, adaptive, and, possibly, tissue/cell context-dependent [4].

Head and neck squamous cell carcinomas (SCCs) are one of the best examples of cancer with infiltrative growth that typically reveals a phenotype of collective cell movement [24]. Indeed, clustered cohort-like cancer cell dissemination appears to be highly efficient in embolizing lymphatic and blood vessels, which are accepted as a major prognostic factor for cancer [25]. We have recently set up an in vitro system for the analysis of the collective cell migration of SCC cells in 3D systems [26]. Using that methodology, we showed that both mesenchymal N-cadherin-expressing cancer cells and cancer-associated fibroblasts cooperate in collective migration of epithelial cancer cells by leading their collective migration. Nevertheless, little is still known about the complexity of collective cell invasion in the heterogeneous intratumoral environment of head and neck SCC, and further work is required in order to fully understand the involvement of phenotypic plasticity of tumor and stromal cells in this phenomenon. FAK over-expression and E-cadherin downregulation are frequent events in tissues of advanced SCC; they are associated with lymph node metastasis in head and neck SCC, and act as key molecules regulating single-cell migration [27,28]. It has also become apparent that the expression or activity of MMP-2, among other proteases, is related to FAK activity and the metastatic potential of head and neck SCCs [13]. Specifically, we uncovered that FAK inhibition in SCC-derived cell lines impairs single cell motility and MMP-2 production, a phenotype that was partially reversed by MMP-2 over-expression. However, the crosstalk of FAK and MMP-2 on the collective migration of SCC cells still remains to be well-defined. To address these issues, we used the in vitro system for the analysis of the collective cell migration in 3D systems [26] and found that in vitro collective SCC cell migration depends on the balance between FAK and MMP-2. The absence of functional FAK blocked cancer cell migration but this constrain could be released by SCC cells expressing high levels of MMP-2 or by cancer-associated fibroblasts, both of them acting as leader cells that push the immobile collective unit towards the ECM. In head and neck SCC tissues, MMP-2 and FAK transcript levels directly correlated with each other and with EMT genes, but only perturbed MMP-2 gene expression was associated with poor outcome independently on FAK expression, the EMT transcriptional phenotype, size of the tumor or disease stage.

## 2. Results

### 2.1. Modulation of Collective Cell Migration by Focal Adhesion Kinase

**Figure 1:**
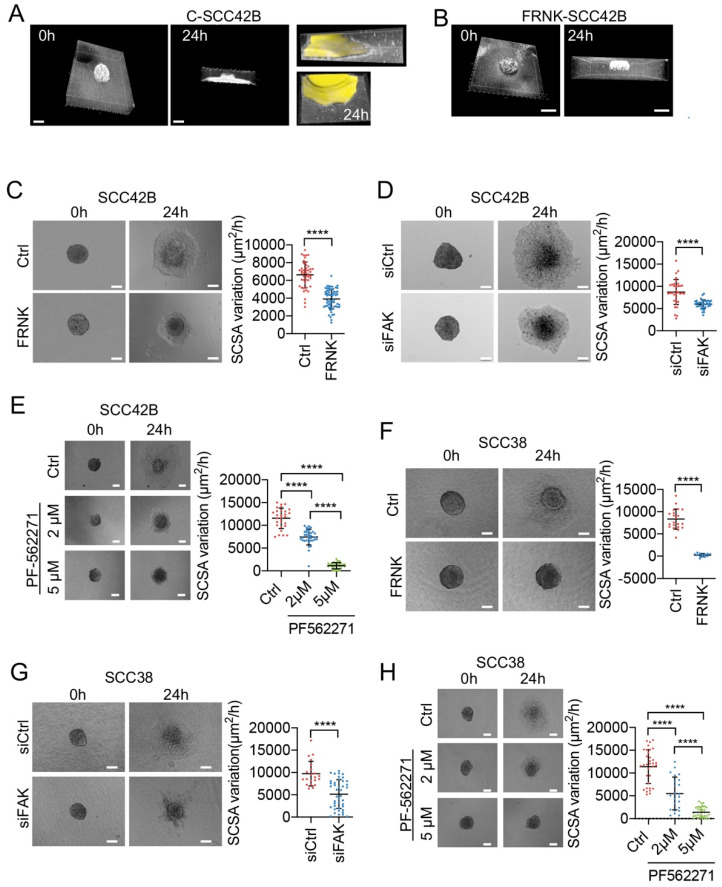
FAK activity is required for efficient collective cell movement of squamous cell carcinoma (SCC) cells. (**A**,**B**) Trajectory and directionality of collective migration of SCC cells in collagen-embedded cell spheroids. Three-dimensional reconstruction of Z-stacked images of spheroids assembled using SCC42B (**A**) or FRNK-SCC42B (**B**) cells and embedded into a collagen matrix. Images of freshly assembled spheroids (0 h) and spheroids after a 24 h migration period (24 h) were captured using confocal reflection microscopy. The resulting images represent a stack of 28 sections (Z step of 2.8 μm) with a total physical length of 140 μm (0 h) or 20 sections with a total physical length of 100 μm (24 h). A yellow pseudocolor and an xz projection (24 h far left pictures) of the same spheroid was used to better visualize the entire spheroid’s area. (**C**–**H**) Representative images from time-lapse movies of SCC42B (**C**–**E**) or SCC38 (**F**–**H**) cells embedded as spheroids into collagen matrix and allowed to migrate for 24 h. Ctrl: cells expressing empty vector (**C**,**F**) or cells treated with PF-562271 vehicle (**E**,**H**); FRNK: FRNK-expressing cells; siCtrl and siFAK: SCC cells after treatment with control or FAK siRNAs, respectively; PF-562271 FAK inhibitor was added to cells at 2 or 5 μM final concentrations (**E**,**H**). Graphics at the right of panels (**C**–**H**) represent variations over time of the spheroid cross-sectional areas (SCSAs) of the spheroids assembled with the indicated cells. For SCSA quantification, at least 10 spheroids from at least 3 independent experiments were used. Scale bars: 100 µm. **** indicates *p* < 0.0001.

As recently reported [26], SCC38 and SCC42B cells assembled as multicellular spheroids in collagen matrix migrate as a collective unit following an outwards and downwards direction as observed upon three-dimensional reconstructions of Z-stacked images taken after 24 h of culture (Figure 1A).

To determine the role of FAK on collective cell invasion, FAK-deficient SCC42B cells were generated by stable expression of the FRNK [29], the noncatalytic COOH-terminal domain of FAK, that functions as a dominant negative inhibitor of FAK autophosphorylation and of tyrosine phosphorylation of focal contacts (Figures S1 and S2A). Cohesive cellular spheroids were properly assembled with FRNK-expressing cells (FR-SCC42B). However, measurement of the variation over time of the spheroid cross-sectional area (SCSA) revealed a significant reduction (1.7-fold reduction in mean SCSA, *p* < 0.0001) in the invasive activity of FR-SCC42B cells compared with FAK-proficient SCC42B cells (C-SCC42B) (Figure 1A–C). We next extended this study by using siRNAs to decrease FAK mRNA and protein expression levels (Figure S2B). FAK-siRNA-treated cells also had a reduced capacity of invasion compared with cells transfected with control siRNA (Figure 1D; 1.45-fold decrease in mean SCSA, *p* < 0.0001). This effect was less potent than that observed in FR-SCC42B cells, likely because of residual FAK expression by incomplete silencing (Figure S2B). Subsequently, we used a pharmacological approach in which we tested the effect of PF-562271, an ATP-competitive reversible FAK inhibitor (Figure S2C). Figure 1E shows a dramatic decrease in cell invasion observed in SCC42B cells exposed to 2 and 5 µM of PF-562271 for 24 h (1.6 and 10-fold decrease in mean SCSA, respectively, *p* < 0.0001) compared with control cells.

The effect of FAK on collective cell invasion was not a cell-line-specific function. In addition to SCC42B cells, we also analyzed SCC38 cells which have greater invasive activity than SCC42B [26] and express higher levels of FAK and pFAK protein (Figure S2). FRNK-induced inhibition of FAK activity in SCC38 cells (FR-SCC38) (Figure S2) also impaired collective invasion (37-fold decrease in mean SCSA, *p* < 0.0001, Figure 1F) compared with control cells (C-SCC38). FAK-siRNA and PF-562271 also decreased functional FAK levels (Figure S2) and collective cell invasion of SCC38 cells by 1.9-fold and 2–7-fold, respectively (*p* < 0.0001) (Figure 1G–H). Finally, FRNK, PF-562271 and FAK-siRNA also decreased collective cell invasion of an additional SCC cell line, SCC40 (Figure S3). These data suggest that FAK has a relevant role in the collective invasion of SCC cells.

### 2.2. Cancer-Associated Fibroblasts Derived from Human SCCs Promote and Lead the Invasive Front of FAK-Deficient SCC Cells in an MMP-2 Independent Mode

**Figure 2:**
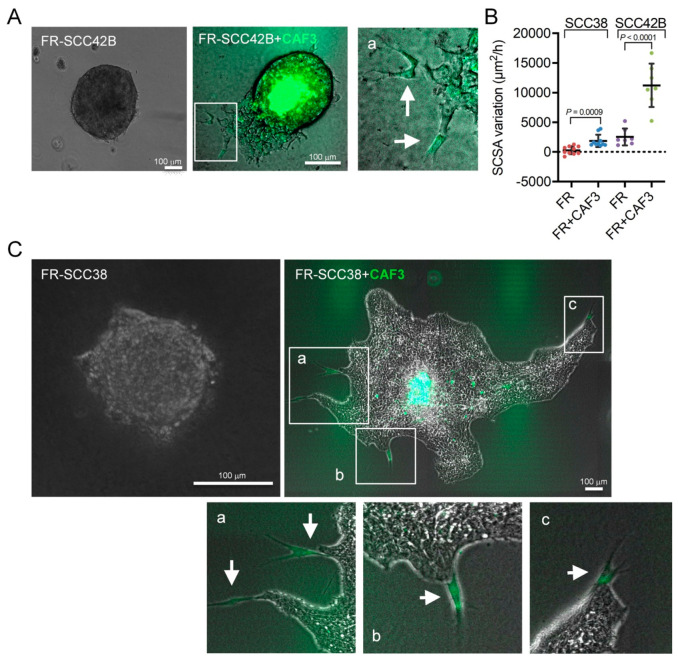
Cancer-associated fibroblasts (CAFs) promote collective invasion of FRNK-SCC cells by acting as leaders of the invasive fronts. (**A**,**C**) Representative images of spheroids assembled with FR-SCC cells or with mixed populations of SCC cells and CAFs as indicated and incubated for 20 h (**A**) or 6 days (**C**). CAFs were labeled with green 5-chloromethylfluorescein diacetate (CMFDA) to allow their tracking over time. Magnified images in panel (**A**) (**a**) and (**C**) (**a**–**c**) are shown to better visualize the leader CAFs (denoted by white arrows) at the tip of the invasive cell tracks. (**B**) Variations over time of the spheroid cross-sectional area (SCSA) of the spheroids assembled with the indicated cells.

Several studies have shown that migratory cells can promote the invasion of immobile cells. We and others have shown that cancer-associated fibroblasts (CAFs) promote the invasive capacity of cancer cells acting as leaders of the invasive fronts of collective cell units [26,30,31]. Thus, we raised the question whether CAFs could counteract the effect of FRNK on the invasive capacity of SCC cells. To this end, we used two established primary cultures of CAFs (CAF1 and CAF3) derived from human SCC tumors [26]. Mixed spheroids were assembled by using equal amounts of FR-SCC42B cells and CAFs to explore how the interaction between cancer and stromal cells influence cell invasion. To identify CAFs, these cells were labeled with green 5-chloromethylfluorescein diacetate (CMFDA). As shown in Figure 2A, in contrast to FR-SCC42B spheroids, mixed spheroids did invade the collagen matrix forming finger-like cell tracks led by one CAF and followed by FR-SCC42B cells. Similar behavior was detected in the FR-SCC38 + CAF spheroids in which we observed the formation of very long cell tracks led by a single CAF even after 6 days in culture (Figure 2C). Moreover, the velocity of the invasion of the cells was significantly increased in the presence of CAFs (Figure 2B). Thus, in the absence of functional FAK, CAFs can pull immobile SCC cells towards the ECM.

**Figure 3:**
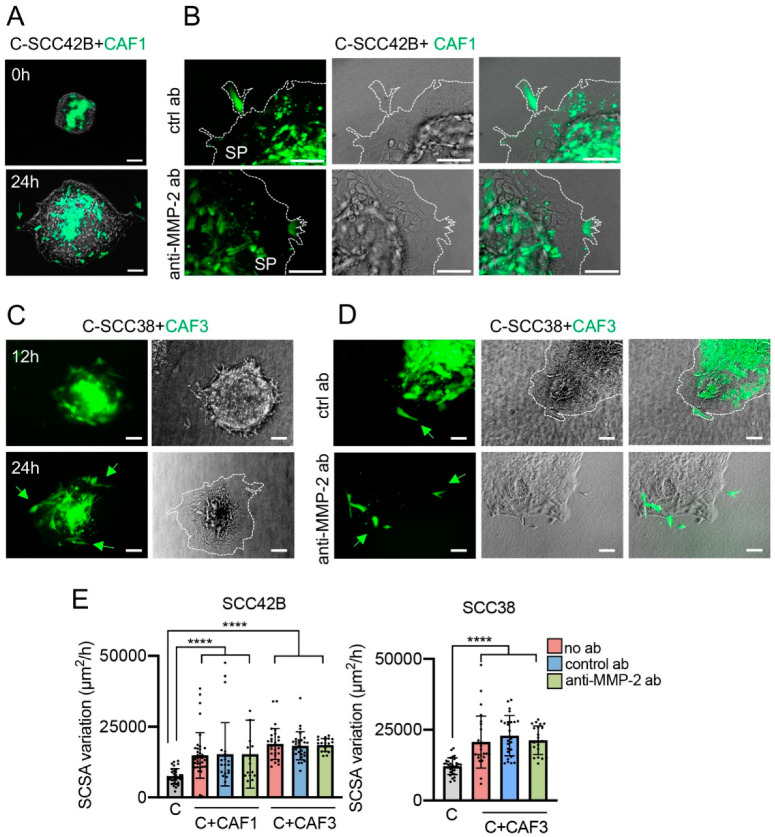
Effect of anti-MMP-2 antibodies on the collective migration of mixed SCC + CAF spheroids. (**A**,**D**) Representative images of mixed spheroids containing control (C-SCC42B or C-SCC38) cells and CMFDA-labelled CAFs and incubated for 0, 12 or 24 h in the absence of antibodies (**A**,**C**), or in the presence of control anti-SDHB (ctrl ab) or anti-MMP-2 antibodies (**B**,**D**). In panels (**B**–**D**), bright field and fluorescence images of the same spheroids are shown. (E) Variations over time of the SCSA of the spheroids assembled with C-SCC or C-SCC + CAF. Data are presented as mean ± standard deviation from 3 individual experiments; at least, 15–30 spheroids were used for quantifications. SP, spheroid. Scale bars, 100 µm. **** indicates *p* < 0.0001.

In addition to the effect of CAFs on FAK-deficient cells, CAFs also promoted migration of FAK-proficient cells as we recently reported [26]. Here, these data were confirmed. As shown in Figure 3, CAF1 and CAF3 (labeled with green CMFDA) significantly increased the collective migration of C-SCC42B cells by 2- and 2.3-fold, respectively (*p* < 0.0001), and took the front positions of the cell tracks. In the case of SCC38 cells, while migration of C-SCC38 + CAF1 spheroids did not differ from that of C-SCC38 spheroids, in agreement with our recent report [26], we did observe increased migration of C-SCC38 cells when assembled into spheroids with CAF3 (1.7-fold, *p* < 0.0001). In line with the phenotype of CAFs in the C-SCC42B + CAF spheroids, CAF3 cells migrated to the periphery of the C-SCC38 + CAF3 spheroids. Moreover, single CAF3 cells, adhered to C-SCC38 cells, were detected in the fronts of the mixed spheroids although in this case we did not observe formation of the long cell tracks headed by CAF3 that had been detected in mixed FR-SCC38 + CAF3 spheroids.

It has been established earlier that the effect of CAFs in promoting collective cancer cell migration is associated with their ability to remodel the ECM and create tracks for cancer cells [32,33,34]. Clustering of integrins and cell–cell interactions are involved in the activation of matrix metalloprotease (MMP) proteins, as MMP-2. In this sense, we previously showed that a crosstalk between FAK and the MMP-2 ECM protease is operational in the SCC cells used in the present study [13]. Thus, we tested whether the blockage of the MMP-2 protein by anti-MMP-2 antibodies would inhibit the effect of CAFs on SCC cell invasion. Analysis of the SCSA variation over time revealed that the rate of cell migration of mixed C-SCC42B + CAFs spheroids in the presence of anti-MMP-2 antibody was similar to that elicited in the presence of antibody against the mitochondrial SDHB protein, used as control (Figure 3E). However, inspection of cells’ positions in the spheroids revealed that, although CAFs took the front positions, the longer cell tracks protruding from the spheroid led by a single CAF were not detected in the presence of anti-MMP-2 antibody (Figure 3A,B), thus suggesting that MMP-2-induced remodeling of the ECM provides an advantage for the CAFs’ leadership capacity in collective cell migration. Invasion of mixed C-SCC38 + CAF3 spheroids, however, was not altered by anti-MMP-2 antibodies. These data suggest that CAFs’ promotion of collective invasion of SCC cells cannot be simply attributed to their ability to secrete the MMP-2 metalloprotease.

### 2.3. MMP-2 Promotes Collective Cell Invasion and Counteract the Effect of FAK Inhibition

**Figure 4:**
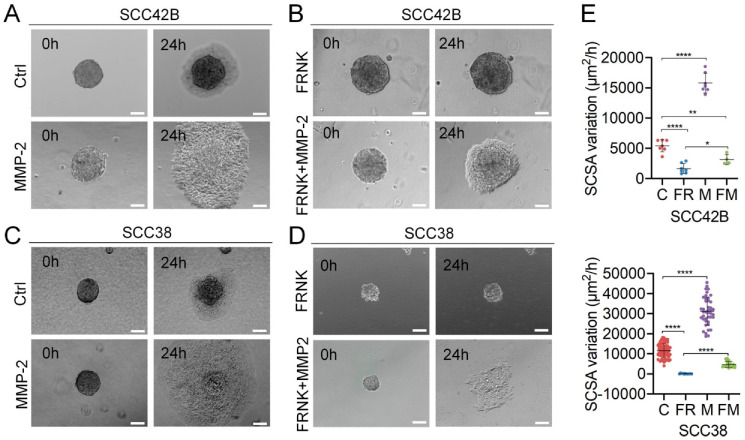
MMP-2 promotes collective cell invasion of FAK-deficient and proficient SCC cells. (**A**–**D**) Spheroids were assembled with the indicated SCC cells infected with empty vector (Ctrl) or overexpressing FRNK, MMP-2 or FRNK + MMP-2 and incubated for 0 or 24 h. (**E**) Variations over time of the SCSA of control (C), FRNK (FR), MMP-2 (M) or FRNK + MMP-2 (FM) spheroids. Data are presented as mean ± standard deviation from 3–5 individual experiments. Scale bars, 100 µm. * indicates *p* < 0.01, ** indicates *p* < 0.001, **** indicates *p* < 0.0001.

Next, we tested whether the MMP-2 matrix metalloprotease, which is downregulated in FR-SCC cells [13] (Supplementary Figure S4) and it is involved in the FAK-induced migration of SCC cells under 2D culture conditions [13], could also modulate collective cell invasion. To this end, we generated SCC42B and SCC38 cells overexpressing MMP-2 (M-SCC42B and M-SCC38 cells) (Figure S4). As shown in Figure 4, MMP-2 over-expression strongly increased (3- and 2.7-fold in SCC42B and SCC38 cells, respectively; *p* < 0.0001) collective cell invasion as compared with control cells. In addition, the stable over-expression of MMP-2 in FRNK-expressing cells partially rescued the inhibitory effect of FRNK in collective cell invasion. Indeed, higher rate of collective cell migration was observed in FRNK + MMP-2-SCC cells (FM-SCC) cells as compared with FR-SCC cells (2-fold (*p* = 0.018) or 33-fold (*p* < 0.0001) increase in SCC42B and SCC38 cells, respectively) (Figure 4). For comparison, three-dimensional reconstructions of Z-stacked images captured with confocal reflection microscopy of spheroids assembled using C-, FR-, M, and FM-SCC42B cells after a 24 h migration period are shown in Supplementary Figure S5.

**Figure 5:**
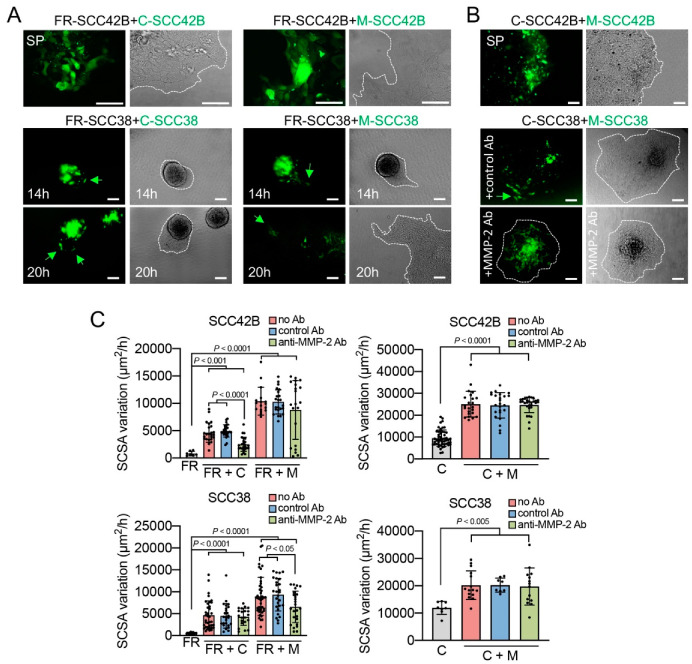
Mixed spheroids containing unlabeled FR-SCC cells plus either CMFDA-labelled C-SCC or CMFDA-labelled M-SCC cells were incubated for 14 or 20 h in the absence of antibodies or in the presence of control (anti-SDHB) or anti-MMP-2 antibodies. (**A**,**B**) Representative bright field and fluorescence images of the indicated mixed spheroids. Dashed white lines are indicated in the bright field images to delineate the periphery of the spheroid. Green arrows point to CMFDA-labelled SCC cells located at the front invasive positions. (**C**) Variations over time of the SCSA of the indicated spheroids. C: control cells, FR: FR-SCC cells, FR + C and FR + M: mixed spheroids containing unlabeled FR-SCC cells plus CMFDA-labelled C-SCC or CMFDA-labelled M-SCC cells, respectively. C + M: mixed spheroids containing unlabeled C-SCC cells plus CMFDA-labelled M-SCC. Data are presented as mean ± standard deviation from 3 individual experiments. Scale bars, 100 µm.

As SCC tumors have a high degree of cellular heterogeneity, we tested whether, similarly to CAFs, the presence of M-SCC or C-SCC cells in FR-SCC multicellular spheroids could release FR-SCC cells from their restrained migratory capacity. To this end, mixed spheroids of CMFDA-labelled-M-SCC (or C-SCC), plus FR-SCC, cells were assembled and allowed to migrate for 20 h. As shown in Figure 5, both M-SCC and C-SCC cells allowed collective cell migration of the otherwise immobile FR-SCC cells, an inductive effect that was significantly higher with M- than C-SCC38 cells, as expected (Figure 5). Significantly, fluorescence microscopy images revealed that the CMFDA-labelled cells (C or M cells) moved towards the front of the spheroids but did not detach from FR-SCC38 cells, thus suggesting that FAK-proficient cells pulled FR-SCC38 cells which were mostly localized in the rearguard of the spheroid. Although this phenotype was detected with the two cell lines, SCC38 and SCC42B, differences were observed regarding the distribution of M- or C-SCC cells within the spheroids. Microscopy analysis of mixed SCC42B spheroids revealed that the leading edge was formed by CMFDA-labelled M- or C-SCC42B-multicellular blunted edges but not a long finger-like protrusion with a unique leader invasive cell. This suggests that most CMFDA-labelled M-SCC42B cells emerged from the migrating collective and took the front positions of the invasive spheroid to serve as leaders (Figure 5A). In contrast, fingerlike cell tracks were formed in C- or M + FR-SCC38 spheroids which were led by 1 or 2 CMFDA-labelled M- or C-SCC38 cells. This suggests that only a reduced subset of M- or C-SCC38 cells is able to take the front positions and push the FR-SCC38 immobile cells thus mimicking CAFs’ behavior observed in the FR-SCC38 + CAF3 spheroids. Collectively, the data show that FAK-proficient cells (C-SCC and M-SCC) are able to push FAK-deficient immobile cells but with different strategies depending on the peculiarities of the cellular background and/or the cell-to-cell interactions.

Blockage of secreted MMP-2 by anti-MMP-2 antibody significantly blunted the effect of C-SCC42B cells in the mixed spheroids with FR-SCC42B cells (*p* < 0.0001) (Figure 5). This inhibitory effect was also observed in FR + M-SCC42B spheroids, but it only affected about 20–30% of mixed spheroids, such that statistical significance was not reached. The effect of M-SCC38 cells on mixed FR + M spheroids was also specifically repressed by the addition of the anti-MMP-2 antibody compared with control antibody (1.4-fold decrease, *p* = 0.004). The effects of anti-MMP-2 antibodies on SCSA variation could not be replicated in mixed spheroids of C-SCC + M-SCC cells likely because of the already very high levels of extracellular MMP-2 in the co-cultures that could not be blocked by anti-MMP-2 antibodies. Nevertheless, anti-MMP-2 antibodies prevented migration of M-SCC38 cells (which express lower levels than SCC42B; data not shown) towards the borders of the spheroids (Figure 5B).

### 2.4. Cellular Phenotypic Plasticity Associated with FAK Overexpression in SCC Cells

**Figure 6:**
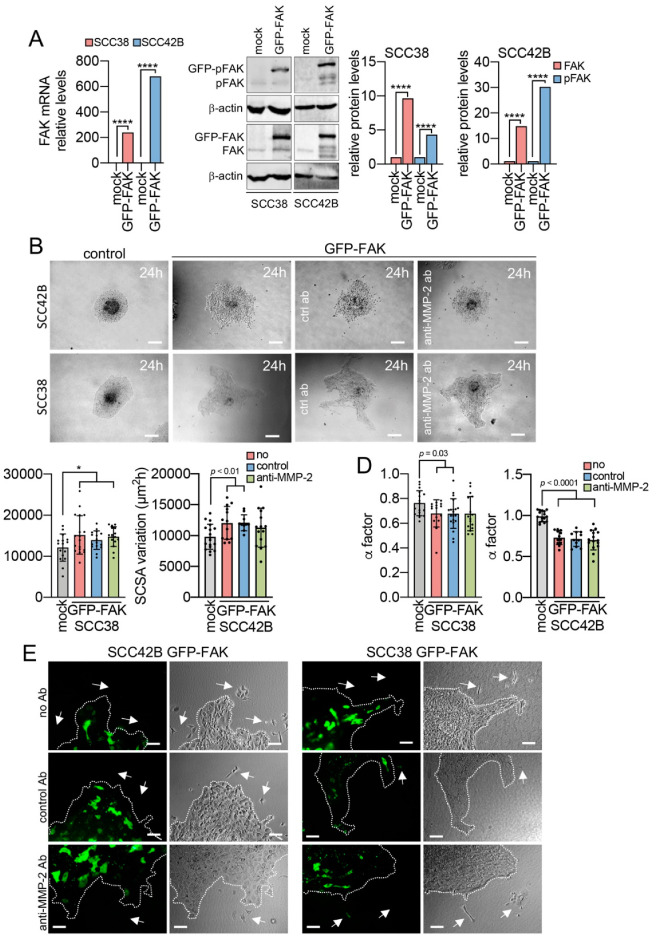
FAK over-expression not only induces collective cell invasion but favors the emergency of cells that detach from the spheroids. (**A**) Representative data showing the efficiency of GFP-FAK expression in transiently transfected cells. Representative immunoblot analysis of FAK and pFAK in mock- and GFP-FAK-transfected cells is shown in the middle. The membranes were stripped and reprobed with anti-β-actin antibody to assure even loading of proteins in each lane. The ratio FAK or pFAK/β-actin was estimated by densitometry. Graphics show relative quantification of FAK mRNA, and FAK and pFAK proteins in the indicated cells. (**B**) Representative images of spheroids assembled with the indicated cells after 24 h of migration in the absence of any antibody or in the presence of control or anti-MMP-2 antibody. (**C**) Variations over time of the SCSA of the indicated spheroids. (**D**) Variations of the α factor of the indicated spheroids after 24 h of migration. (**E**) Representative bright field and fluorescence images of the indicated spheroids after 24 h of incubation in the presence or absence of the indicated antibodies. White dashed lines indicate the periphery of the spheroids. White arrows point to cells that escaped from the spheroids and migrated as single cells, most of which were GFP-negative. Scale bars, 100 µm. * indicates *p* < 0.01, **** indicates *p* < 0.0001.

The effect of FAK over-expression was analyzed by deriving SCC42B and SCC38 cells that over-express the fusion protein GFP-FAK (hereafter FAK-SCC cells) by transient transfection with pGFP-FAK plasmid. We assessed efficiency of transfection by quantification of: GFP-labelled cells by fluorescence microscopy, total and active FAK (pFAK, the autophosphorylation of FAK on Y397 is used as readout of FAK activation) expression by Western blot, and FAK mRNA levels by qPCR. Fluorescence microscopic analysis revealed that about 50% of SCC42B and SCC38 cells were efficiently transfected (data not shown). At the mRNA level, the expression of the FAK-encoding gene was robustly increased in FAK-SCC cells, especially in SCC42B cells. Similar increase was detected at the protein levels of both, total FAK and its activated form, pTyr397 FAK, in FAK-SCC cells compared with mock-transfected cells (Figure 6A).

FAK-SCC or mock cells were then assembled into cellular spheroids that were allowed to migrate for 24 h. It should be noted that the GFP-FAK spheroids had a heterogeneous cell population consisting of GFP-positive cells, which correspond to cells efficiently transfected and expressing high levels of GFP-FAK, and GFP-negative cells, which are cells that had not been transduced. Significantly, these heterogeneous FAK-SCC spheroids had a subtle increase (1.2-fold increase, *p* = 0.01) in invasive activity compared mock-transfected cells (Figure 6B,C). More remarkably, what this assay revealed was that while spheroids with mock-transfected cells maintained a rounded morphology over time indicating that cells migrated isotropically, the spheroids assembled with FAK-SCC cells adopted an irregular shape over time due to the extension of cell strands from the borders of spheroids. To quantify this phenomenon, we used the shape factor (α) [34] that measures the variations of the length of the contour of the spheroids. The α factor, which may range from 0 (when the spheroid displays a very irregular interface) to 1 (when the spheroid is a perfect circle), was significant lower in FAK-SCC38 and FAK-SCC42B spheroids compared to mock spheroids in a 24 h period of migration (Figure 6D). In addition to this, less cohesiveness between cells was detected in FAK-SCC spheroids in comparison with spheroids of mock-transfected cells. Accordingly, cells emerged at the periphery of FAK-SCC spheroids that lost cell-to-cell contacts and detached from the spheroid migrating individually, a phenotype not observed in spheroids with mock-transfected cells (Figure 6E). Surprisingly, fluorescence microscopic analysis showed that both GFP-labelled and unlabeled cells were equally distributed at the invasive fronts of the spheroids and that cell detachment from the spheroids mostly concerned GFP-unlabeled cells, not SCC cells expressing GFP-FAK, which were detected very occasionally.

As MMP-2 expression is regulated by FAK [13], we sought to determine whether anti-MMP-2 antibodies could blunt the effects of FAK over-expression. Very subtle reduction in SCSA variation over time was detected in GFP-SCC42B spheroids by anti-MMP-2 antibodies in comparison with untreated cells. Lessening of the α factor in GFP-FAK-SCC38 cells was barely mitigated with anti-MMP-2 antibodies (Figure 6C,D). Fluorescence microscopy analysis revealed that cells escaping from the spheroids were still detected in FAK-SCC cells incubated with anti-MMP-2 antibodies. Collectively, the data suggest that FAK over-expression not only promotes collective cell migration, in accordance with our previous results, but also promotes phenotypic cellular plasticity inducing a reprograming of non-transduced cells to modify their migratory phenotype via a mechanism in which MMP-2 has a very faint implication, if any.

### 2.5. EMT Plasticity Associated with FAK and MMP-2 Expression

**Figure 7:**
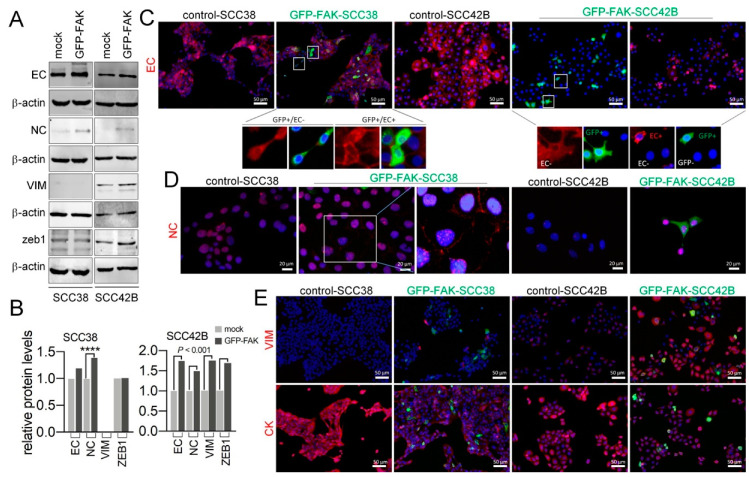
FAK over-expression in SCC cells favors epithelial to mesenchymal transition (EMT) of adjacent non-transduced SCC cells. (**A**) Representative immunoblot of E-cadherin (EC), N-cadherin (NC), vimentin (VIM) and ZEB1 in mock- or GFP-FAK transfected SCC cells. The membranes were stripped and reprobed with anti-β-actin antibody to assure even loading of proteins in each lane. The ratio EC, NC, VIM or ZEB1/β-actin was estimated by densitometry. Quantification of the relative protein levels is shown in (**B**). (**C**–**E**) Representative images of immunostaining for EC, NC, VIM and CK in the indicated cells. Insets in C are shown to highlight that GFP-positive cells at the periphery, but not in the middle, of the migrating nests lose EC immunostaining. The inset shown in panel (**D**) is an over-exposed image to highlight the membrane NC immunostaining in GFP-FAK-SCC38 cells. **** *p* < 0.0001.

Classical EMT-transcriptional program weakens cell–cell junctions between cancer cells inducing individualization of tumor cell migration. At the molecular level, the hallmark of EMT is the upregulation of N-cadherin (NC) followed by the downregulation of E-cadherin (EC). Thus, we asked whether FAK over-expression induced changes in the epithelial phenotypes of the genetically engineered SCC cell lines by analysis of the NC and EC protein levels. Vimentin (VIM) and cytokeratin (CK) levels were also analyzed as mesenchymal and epithelial markers, respectively. Of note, as described in our previous report [35], the SCC38 cell line contains cells that mostly have an epithelial phenotype (express EC and CK and do not express VIM; NC expression is testimonial). The SCC42B cell line, by contrast, contains not only cells with a canonical epithelial phenotype but also a 50% of cells with a hybrid epithelial/mesenchymal phenotype that co-express EC and VIM but not NC. As shown in Figure 7A,B, Western blot analysis revealed that EC did not decrease but slightly increased in both SCC38 and SCC42B cells upon GFP-FAK over-expression as compared with mock-transfected cells. Regarding the mesenchymal biomarker, NC, we did observe significant increase upon GFP-FAK-transfection in both SCC38 and SCC42B cell lines. VIM protein levels also increased upon GFP-FAK over-expression in SCC42B cells but could not be detected by Western blot in mock, nor GFP-FAK-SCC38 transfected, cells. Induction of GFP-FAK expression in SCC42B cells also increased significantly the levels of ZEB1, a key element of the network of transcription factors controlling EMT. Thus, FAK overexpression induces reprograming of cells toward partial EMT.

By using GFP as a marker of FAK over-expression, we next sought to determine whether cells undergoing partial EMT were those with or without FAK over-expression. Immunofluorescence analysis revealed that, globally, EC immunostaining was weaker in the GFP-negative SCC38 and SCC42B cells upon transient transfection with GFP-FAK than in cells transfected with control vector (Figure 7C). In GFP-positive cells, some heterogeneity was observed such that those cells within the tumor nest were mostly EC positive, whereas those at the periphery showed loss of membrane EC immunostaining suggesting that they were undergoing EMT. Interestingly, GFP-positive SCC42B cells located in the tumor nest did not lose EC but even increased its levels in contrast with GFP-negative cells. This heterogeneity may explain the higher levels of EC detected by Western blot analysis in FAK-SCC42B cells.

As indicated above, the mock-transfected SCC38 cell line lacks NC positive cells. By contrast, transfection with GFP-FAK caused the emergence of NC-positive cells which were mostly, although not exclusively, GPF-negative cells. With regard to SCC42B cells, NC membrane immunostaining could not be detected neither in mock nor in GFP-FAK-transfected cells, calling into question whether the increased NC levels detected by Western blot reflected the presence of NC in any intracellular compartment.

A reduced percentage of VIM positive cells was detected in GFP-FAK-transfected SCC38 cells but not in mock-transfected cells. Of note, these data reveal that we had been unable to detect VIM in FAK-SCC38 cells by Western blot because of the limited level of this protein that is likely below the detection limit of the immunoblotting assay. Alike NC, VIM expression was mostly, but not exclusively, detected in GPF-negative cells. The epithelial biomarker, CK, showed weaker immunostaining in GFP-FAK-SCC38-transfected cells but we could not distinguish whether there was a preferential effect in GFP-positive or negative cells. Increased VIM and decreased CK protein levels were detected in all SCC42B cells irrespective of whether they are GFP-positive or negative. Thus, FAK overexpression induced partial EMT with mesenchymal and epithelial characteristics that, although it could affect some cells over-expressing FAK, it was mostly concerned with neighboring GFP-negative cells.

**Figure 8:**
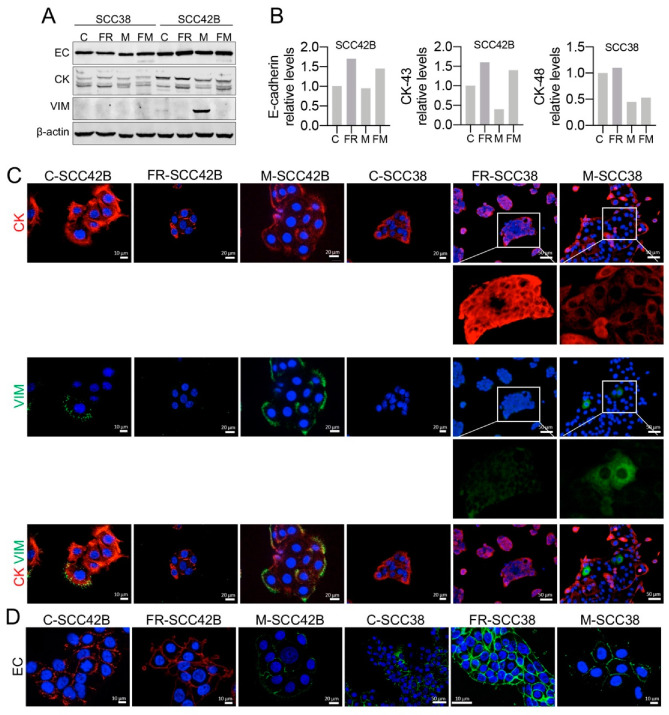
Expression of epithelial/mesenchymal biomarkers in association with FAK/MMP-2 in SCC cells (**A**) Western blot analysis showing expression of EC, CK and VIM in SCC cells infected with empty vector (**C**) or in SCC cells expressing FRNK (FR), MMP-2 (M) or FRNK + MMP-2 (FM). The membranes were stripped and reprobed with anti-β-actin antibody to assure even loading of proteins in each lane. The ratios EC, CK or VIM/β-actin were estimated by densitometry (**B**). (**C**,**D**) Representative images of immunostaining of the indicated cells for CK (red) plus VIM (green) (**C**) or EC (red or green) (**D**). Insets in panel (**C**) highlight the loss of CK and gain of VIM in M-SCC38 cells compared with FR-SCC38 cells.

Expression of epithelial and mesenchymal biomarkers were also analyzed in FRNK-expressing cells. FRNK-induced inhibition of FAK activity increased the expression of epithelial cell markers (EC and CK) in SCC42B cells but the basal levels of these biomarkers were not further increased in SCC38 cells (Figure 8A). Immunocytochemical analysis revealed that the subcellular distribution of EC was modified in the two cell lines. EC was homogeneously distributed at the plasma membrane of FR-SCC38 and FR-SCC42B cells such that the clusters in the form of punctate or linear aggregates, typically found in control cells, were not readily detectable. In addition, immunostaining was detected along the whole perimeter of the FRNK-expressing cells including the surface free of cell-to-cell contacts, whereas it was barely detected at the free surface of control cells (Figure 8C). VIM immunocytochemical analysis revealed the absence of VIM positive cells in FR-SCC42B cells which is consistent with the FRNK-induced decrease in the already low levels of this protein detected by Western blot in SCC42B cells (Figure 8A). These data suggest that SCC cells with non-functional FAK either retain expression of epithelial transcripts or switch from a mesenchymal to a more epithelial phenotype.

The epithelial/mesenchymal markers were also analyzed in M- and FM-SCC cells. MMP-2 over-expression did not modify expression of EC (Figure 8) nor alter its subcellular distribution (data not shown) in both SCC42B and SCC38 cells. However, MMP-2 over-expression decreased the levels of CK in SCC42B and SCC38 cells. Immunocytochemical and Western blot analysis of VIM revealed that MMP-2 over-expression in SCC42B cells strongly increased the proportion of cells co-expressing VIM in addition to CK (Figure 8). Furthermore, MMP-2 over-expression in SCC38 cells caused the emergence of cells co-expressing CK and VIM as detected by immunocytochemical analysis (Figure 8C), although VIM did not reach high enough levels to be detected by Western blot analysis (Figure 8A). Similar results were observed in FM-SCC38 and FM-SCC42B cells (Figure 8). Thus, MMP-2 over-expression not only increased collective cell migration but directly induced an increase in the population of cells undergoing partial EMT distinguished by co-expression of epithelial (EC and CK) and mesenchymal (VIM) biomarkers via a FAK-independent mechanism.

**Figure 9:**
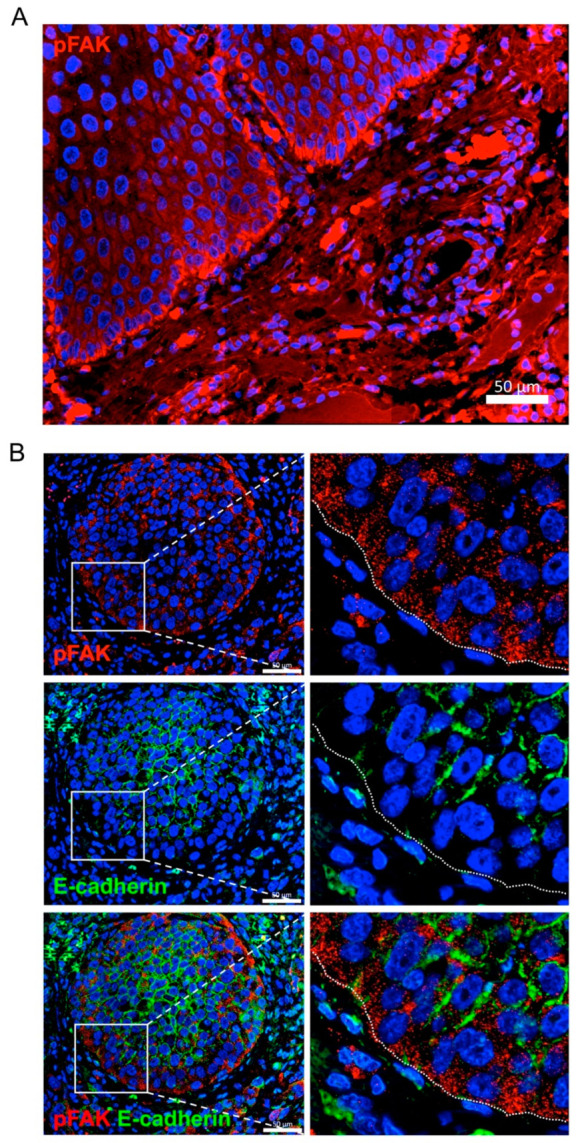
pFAK is highly expressed at the borders of cancer cell nests matching with areas of loss of E-cadherin expression. (**A**) Representative image of pFAK immunofluorescence in head and neck SCCs showing high expression levels at the border of the tumor. (**B**) Double immunofluorescence labeling of a head and neck SCC tissue for E-cadherin (red) and pFAK (red) showing that pFAK is predominantly expressed at the border of the cell nest where E-cadherin is lost. Insets show the boundary between the tumor nest and the surrounding stroma (dashed white line). Scale bars, 50 µm.

To determine whether our in vitro observations could have an in vivo correlate, we analyzed the distribution of active FAK (pFAK) and EC by immunofluorescence in human head and neck SCC. Consistent with the in vitro data, we found that pFAK immunostaining was enriched in the invasive leading edges of cell nests where EC expression was found to be lost (Figure 9). In addition to pFAK, MMP-2 was also found to be more highly expressed in the most invasive areas of the head and neck SCCs (Figure S6).

### 2.6. FAK and MMP-2 mRNA Expression in Head and Neck SCC and Clinicopathologic Variables

**Figure 10:**
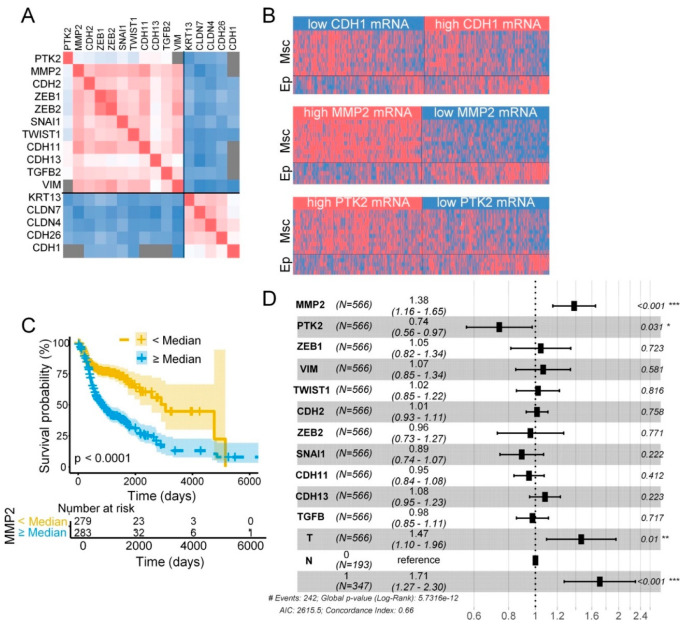
High *MMP2* mRNA levels are significantly associated with poor prognosis independently of EMT transcriptional signature or *PTK2* mRNA levels. (**A**) A heatmap showing positive (red) and negative (blue) correlations of mRNA levels of *PTK2*, *MMP2*, and the indicated mesenchymal (*CDH2, ZEB1, ZEB2, SNAI1, TWIST1, CDH11, CDH13, TGFB2, VIM*) and epithelial (*KRT13, CLDN7, CLDN4, CDH28, CDH1*) biomarkers in head and neck SCCs included in the TCGA dataset (n = 566 tumors). (**B**) Graphic representation of the epithelial/mesenchymal gene expression signature of head and neck SCC included in the TCGA dataset. Tumors were dichotomized into high (red) or low (blue) gene expressors taking into account whether the mRNA levels of *MMP2, PTK2, CDH1* and the epithelial (Ep) genes, *CLDN7, CLDN4, CDH26, KRT13*, or mesenchymal (Msc) genes, *CDH2, ZEDB1, ZEB2, SNAI1, SNAI2, TWIST1, CDH11, CDH13, TGFB2*, and *VIM*, were, respectively, above or below the median value of mRNA levels of the corresponding gene. (**C**) Kaplan–Meier curves for head and neck SCC patients (TCGA database) according to the *MMP2* mRNA levels (median value = 12.3). The 95% confidence intervals (shaded areas) are also represented; permutated log-rank *p*-value for the hypothesis testing of equality of curves between groups is also reported. (**D**) Forest plot of primary outcome indicating genes associated with prognosis in head and neck SCCs.

Expression of MMP2, *PTK2* (FAK encoding gene) and a list of epithelial (Ep)/mesenchymal (Msc) biomarkers were further analyzed at the mRNA level in the 566 head and neck SCCs included in the TCGA database. First, we verified that, as predicted from standard EMT models, expression of Ep and Msc genes directly and inversely correlated, respectively, with the transcript levels of the EC- coding gene (*CDH1*). We also corroborated that *MMP2*, an accepted Msc biomarker, directly and inversely correlated, respectively, with expression levels of Msc and Ep genes (Figure 10A,B, Table S1).

Analysis of *PTK2* also revealed a significant and direct correlation with the Msc biomarkers and inverse correlation with the Ep biomarkers (Figure 10A,B, Table S1). In addition, mRNA levels of *MMP2* and *PTK2* were also directly and significantly correlated. However, neither *MMP2* nor *PTK2* transcript levels correlated with those of *CDH1*. These data suggest that FAK and MMP-2 tend to be co-over-expressed in head and neck SCCs and moderately or strongly associate, respectively, with a Msc cell phenotype.

Table 1 shows the association of *PTK2* mRNA levels with clinicopathologic variables. We previously reported that FAK protein expression was higher in head and neck SCC that developed lymph node metastasis than in those without nodal metastasis [27]. Analysis of *PTK2* mRNA levels in the 566 head and neck SCCs included in the TCGA database indicated that higher transcript levels of *PTK2* were associate with nodal metastases (*p* = 0.005). No other statistically significant relationships were observed between *PTK2* mRNA levels and other clinical variables. *MMP2* transcript levels were significantly associated with nodal metastasis (*p* = 0.021), T stage (*p* < 0.0001) and disease stage (*p* = 0.007).

No association between *PTK2* transcript levels and disease outcome was observed. By contrast, patients with high *MMP2* transcript levels had a shorter overall survival (OS) compared to patients with low levels (median, 2.3 vs. 8.5 months, *p* < 0.0001) (Figure 10C). This could be related to the *MMP2*′s relationship with mesenchymal phenotype given that patients with high tumoral transcript levels of other Msc biomarkers such as *CDH2*, *ZEB1/2*, *SNAI1, TWIST1*, *CDH11*, *CDH13*, *TGFB,* and *VIM* also had significant association with shorter OS than patients with low levels (Figure S7). Thus, we tested the associative power of *MMP2* against the other Msc biomarkers, T and N in a multivariate Cox analysis (Figure 10D). Perturbed *MMP2* gene expression was the only molecular biomarker independently associated with poor outcome (hazard ratio 1.38, 95%CI 1.16–1.65, *p* < 0.001) together with size of the tumor (HR 1.47, 95%CI 1.10–1.96, *p* < 0.01) and lymph node metastasis (HR 1.71, 95%CI 1.27–2.30, *p* < 0.001).

## 3. Discussion

There is little information about how factors governing metastasis and collective cell movement in human SCCs intercommunicate with each other. During metastasis development, information from the cellular microenvironment, including cell–cell and cell–ECM interactions, provides cues that coordinate cancer cell phenotypic plasticity and cell migration. Here, we provide evidence that collective migration of SCC cells requires active FAK. However, this requirement can be circumvented by the presence of CAFs in the tumor environment or by the presence of a divergent group of cancer cells that over-express MMP-2. These findings underscore the importance of the tumor heterogeneity and their diverse inputs governing metastatic capabilities of cancer cells, which can have profound clinical effects.

Thus far, the role of FAK on collective cell migration in head and neck SCCs has not been well-defined. Here, we present several pieces of evidence supporting the relevant role of FAK activity in migration of collective cell units. Cell-based assays showed that decreasing FAK activity abolished collective cell migration, which is consistent with data obtained in other cell types [14]. Immunohistochemical analysis of head and neck SCC tissues revealed the presence of activated FAK located in the invasive fronts of tumor cell nests. In addition, we and others showed that higher FAK protein levels in tissue samples were significantly associated with the presence of lymph node metastasis which are typically colonized by embolization of clustered cancer cells [26,36,37]. Our data argue that FAK protein deregulation likely occurs at the mRNA level given that not only high FAK protein levels but also high FAK transcript levels in head and neck SCCs associate with lymph node metastasis. Thus, it is tempting to speculate that FAK activity has a relevant role in cancer expansion to regional lymph nodes via regulation of the collective migration of cancer cells. Although these findings could have important clinical implications, the levels of FAK protein or transcript in tumor tissues did not associate with poor disease prognosis, suggesting that the relationship between the FAK and tumor cell invasion within the tumor ecosystem is more complex. We provide here some clues that could help to understand this complexity.

Collectively migrating cells exhibit homotypic cell–cell interactions, but a less-appreciated dimension is the contribution of heterotypic cell–cell interactions. Accumulating studies implicate CAFs in cancer metastasis through their remodeling of the ECM and release of large amounts of ECM proteins and soluble factors providing guidance for collective invasion of the SCC cells [30,31,32,33]. More recently, heterotypic adhesion involving N-cadherin at the CAF membrane and E-cadherin at the cancer cell membrane have been shown to lead to CAF-led collective cell migration [31]. By the assembling of mixed cell spheroids composed by SCC and CAFs, we recently confirmed the cancer cell–CAF interactions [26]. We show here that the FAK requirement for efficient collective invasion of SCC cells can be circumvented by the presence of CAFs. Indeed, CAFs were able to adhere to FRNK-expressing cells, take the leader positions in the spheroids, and pull the immobile F-SCC cells toward the collagen matrix forming long invasive finger-like protrusions. Interestingly, the cell protrusions were always led by a single fibroblast. These data not only confirmed the previously described leadership role of CAFs in collective cell invasion but revealed that cancer cells harboring FAK perturbations can migrate collectively in the tumor ecosystem containing “trailblazer” CAFs. On another hand, the leadership behavior of CAFs is maintained even when SCC cells are not capable of migration due to the lack of functional FAK.

In addition to CAFs, leader positions of collectively migrating units can be supplied by cancer cells that over-express MMP-2 whenever they are included in spheroids containing either FAK-proficient or FAK-deficient cells. However, in contrast to CAFs, MMP-2-over-expressing cells did not promote the emergence of finger-like tracks in the mixed spheroid suggesting that all outer border MMP-2-over-expressing cells are equally competent to lead collective migration. Thus, molecularly heterogeneous sets of SCC cells move as one functional unit, such that the more migratory cells promote the invasion of less mobile cells. MMP-2 over-expression in FAK-deficient cells can also shift immobile cells into migrating cells. Collectively, the data suggest that FAK activity in the rear positions of the collective cell units is not strictly required for cancer cell invasion and that tumor heterogeneity may provide the stage for promoting the dissemination of cells of different clonality and function within one functional unit even in the absence of clones with cancer-specific genetic perturbations.

Our study also revealed that although impairment of FAK activity in SCC cells abrogated collective movement it did not induce the detachment of individual cells from the collective unit. In fact, SCC cells with decreased FAK activity preserved the epithelial cell phenotype (SCC38 cells) or even seemed to transit from a mesenchymal to an epithelial phenotype (SCC42B cells). In agreement with these data, FAK overexpression not only facilitated collective cell migration but also induced partial EMT. Remarkably, this phenotypic plasticity concerned some cells over-expressing FAK but mostly neighbor cells expressing basal levels of FAK. The presence of FAK-overexpressing cells in the SCC spheroids also promoted the emergence of cells that detached from the collective units and migrated individually, most of them lacking E-cadherin expression. Thus, FAK-overexpressing cells are transmitting signals to other cells to induce partial EMT. MMP-2 does not seem to play a relevant function in this cellular plasticity given that anti-MMP-2 antibodies do not prevent phenotypic cellular transformation. Further investigations are needed to unravel the mechanisms involved in this phenomenon.

On another hand, the spheroids containing FAK-over-expressing cells invaded the collagen matrix slightly faster than those of mock-transfected cells. As we used transient transfections to over-express FAK, we cannot assess whether FAK promotion of cell invasion could reach similar levels to that observed by MMP-2 over-expression. Nevertheless, our approach gave us the opportunity to compare side-by-side the behavior of cells over-expressing FAK with those that do not. The most intriguing observation was that subpopulations of front cells emerged from the migrating collective to serve as leaders forming finger-like structures which seemed to facilitate the migration of followers. Interestingly, the fastest migrating cells were not those over-expressing FAK. Collectively, our data led us to conclude that FAK signaling induces an exchange of information within the cell collective via released molecules and/or transmission of forces through cell–cell contacts that, either directly or indirectly, induce greater collective cell invasion and also partial EMT of neighbor cells, some of them escaping from the collective unit and migrating individually. Although MMP-2 over-expression also promotes EMT in the SCC cells, its activity seems to play a scarce role in FAK-induced phenotypic plasticity since the effects of FAK over-expression are not reversed by anti-MMP-2 antibodies. Thus, our data on FRNK cells show that FAK is required by collective cell invasion via, at least in part, MMP-2 activity, but its overactivation promotes cellular phenotypic heterogeneity via additional, still undefined, signaling.

In agreement with our in vitro data, increased FAK transcript levels in the TCGA cohort of head and neck SCCs correlated with the activation of the transcriptional program governing EMT. In addition, active FAK (pTyr391-FAK: pFAK) in head and neck SCC tissues was mainly found at cancer cells that lacked/had reduced levels of membranous E-cadherin. Although E-cadherin protein levels were not modified by inhibition of FAK activity in SCC cells, we did find that this protein distributed uniformly at the adhered plasma membranes and did not form the finite size clusters detected in FAK-proficient cells. Thus, it is possible that FRNK-induced alterations of E-cadherin supramolecular organization is also preventing effective cell migration. The size, distribution, and lateral dynamics of E-cadherin clusters are indeed expected to impact on adhesive forces and tensile force transmission locally [38,39]. Thus, our data and other published findings highlight the fact that the relationship between the two structures, focal adhesions and adherens junctions, is extremely complex and warrants additional biochemical and microscopic studies to clarify the interrelationships between them and their potential implications in cancer metastasis.

Collectively, the present work unveils that intratumor cellular heterogeneity has a profound impact on the collective cell migration of SCC cells: the presence of “trailblazer” CAFs, of divergent populations of cancer cells, or the balance between the expression levels of FAK and MMP-2 in the same cancer cell population modulates collective cell migration.

Thus, which is the molecular alteration with a predominant role in the metastatic behavior of human tumors? Our data revealed that up-regulation of *PTK2* and *MMP2* genes correlated with the expression of mesenchymal genes, which are expressed as much in cancer cells that fully or partially completed an EMT program as in stromal mesenchymal cells; that high FAK or MMP-2 mRNA levels were correlated with nodal metastasis; that MMP-2 mRNA levels are also significantly associated with tumor size and disease stage. However, perturbed MMP-2 gene expression was the only molecular biomarker independently associated with poor outcome independently on the EMT transcriptional biomarkers, tumor size or disease stage. The implication of our findings is that future therapeutic targeting of the invasion cascade will require taking this tumor heterogeneity and migratory plasticity of cancer cells into consideration. Pending prospective validation studies, we suggest that molecular targeted therapy using FAK inhibitors in combination with MMP-2 inhibitors may help control tumor progression and prolong progression-free survival.

## 4. Materials and Methods

### 4.1. Cell Culture, Transfections and Treatments

The established human squamous cell carcinoma (SCC)-derived cell lines were kindly provided by Dr R Grenman (University Central Hospital, Turku, Finland) [25]. Cancer-associated stromal fibroblast-like cell lines were derived from surgically removed SCCs arising at the larynx (CAF1) or oral cavity (CAF3). All cell lines were grown as previously described [35] and were periodically tested for human pathogens and mycoplasma infection. To avoid cross-contamination and phenotype changes, the cell lines have not been maintained in long-term cultures. All cells used in this study were maintained as frozen stocks and cultured for 2 to 4 weeks only before use in the experiments. Array CGH had been used to characterize genome-wide DNA copy number alterations in these cell lines and authenticate them. This analysis had revealed the presence of an overall pattern that is broadly consistent with the literature in head and neck squamous cell carcinomas. Authentication of these cell lines based on morphology and growth curve analysis was performed regularly, and no phenotype changes were observed through the duration of this study. Short tandem repeat (STR) profiling of the cell lines revealed that there is no match with publicly available profiles of other cell lines, that the cell lines are unique and are not cross-contaminated or misidentified. All methods were carried out in accordance with the approved guidelines of our institution.

siRNA duplex oligonucleotides were purchased from Dharmacon Research (Lafayette, CO, USA). The targeted sequences for FAK siRNAs were: GAUAGUGGACAGUCACAAA, CCAGUUUACUGAAGAUAAG, UUUCUUCUAUCAACAGGUG. siCONTROL nontargeting pool (Dharmacon) was used as control siRNA. pGFP-FAK plasmid was a gift from Kenneth Yamada, (Addgene plasmid #50515; http://n2t.net/addgene:50515; RRID:Addgene_50515) [40]. PF-562271 (Selleckchem, UK) FAK inhibitor was used at 2 or 5 μM final concentration.

Cell lines stably transfected with FRNK, MMP-2 or both genes were generated as previously described [13]. For siRNA experiments, cells were transfected with 50 nM siRNA using Lipofectamine RNAiMAX (Invitrogen, Thermo Fisher, Waltham, MA, USA). Protein analyses revealed a substantial inhibition of FAK expression 48–72 h after transfection with FAK-siRNAs. The siRNA-transfected cells were used for subsequent experiments within that interval of time. Transient transfection with pGFP-FAK was performed with Lipofectamine 3000 (Invitrogen, Thermo Fisher), according to the manufacturer’s protocol at a final concentration of 4 μg of plasmid DNA.

### 4.2. Reverse Transcription and qPCR

Total RNA from cell lines was isolated with mirVanaTM miRNA Isolation Kit (Invitrogen, Thermo Fisher Scientific) following manufacturer’s instructions. cDNA was synthesized from 1 μg of total RNA using Maxima First Strand cDNA synthesis kit for RT-qPCR (Thermo Scientific). SYBR Green PCR Master Mix (Applied Biosystems, Foster City, CA, USA) was used to analyze the expression of FAK and MMP-2 genes. Each sample was analyzed for peptidylprolyl isomerase A (PPIA) mRNA to normalize RNA input amounts and perform relative quantification. All reactions were performed in triplicate and relative mRNA expression was normalized against endogenous controls using the comparative delta–delta CT method. PCR primers used for the qPCR step were: FAK, forward, 5′-CTTCGGACAGCGTGAGAGAGA-3′ and reverse, 5′-GACGCATTGTTAAGGCTTCTTGA-3′; MMP-2, forward, 5′-TGCTGGAGACAAATTCTGGAGATA-3′ and reverse, 5′-GGATCCATTTTCTTCTTCACCTCAT-3; PPIA, forward, 5′-CATCTGCACTGCCAAGACTGA-3′ and reverse, 5′-TTGCCAAACACCACATGCTT-3′.

### 4.3. Multicellular-Spheres Generation and Migration Analysis

For multicellular spheres generation, 1000 cells were cultured in a spheroid formation media (growth culture medium supplemented with 0.2% methylcellulose) in non-adhesive convex environment for 12 h at 37 °C and 5% CO_2_ as previously reported [26]. Multicellular spheres were mixed with collagen matrix (2.5 mg/mL) and incubated for 30 min at 37 °C prior to microscopic analysis as previously described [26]. For co-culture experiments, cells indicated in the figure legends were labeled with the fluorescent dye CellTracker™ Green CMFDA (5-chloromethylfluorescein diacetate; Thermo Fisher) following manufacturer’s instructions. Then, equal amounts of cells were used for spheres generation. For quantification of collective cell migration, the spheroid areas were measured at each time point using the Zen software to define the spheroid cross-sectional area (SCSA) and calculate its variation over time.

### 4.4. Immunohistochemistry and Immunofluorescence

Formalin-fixed, paraffin-embedded human tissues obtained from patients surgically treated at the Hospital Universitario Central de Asturias, or tumor-spheroids, were cut into 3 μm sections and mounted on poly-L-lysine-coated slides (DakoCytomation, Glostrup, Denmark). Antigen retrieval was performed by heating 20 min in a pressure cooker with Envision™ FLEX target retrieval solution pH 9. Tissue slides were incubated for 1 h with the following primary antibodies: mouse IgG anti-E-cadherin antibody (Becton Dickinson Transduction Laboratories, Erembodegem, Belgium) at 1:50 dilution, mouse IgG anti-N-cadherin (Dako, Agilent Technologies, Santa Clara, CA, USA) at 1:200 dilution, and mouse IgG anti-pY397 FAK (Cell Signaling Technology Inc., Danvers, MA, USA) at 1:50 dilution. For immunohistochemistry, all the slides were stained simultaneously in an automated horizontal slide-processing system (Dako Autostainer Plus) as previously described [26]. Negative controls with either an omission of the primary antibody or with a normal mouse IgG (Santa Cruz Biotechnology, Inc., Dallas, TX, USA) in the primary incubation were also included. The slides were digitalized on a Leica SCN400F scanner and analyzed with the SlidePath Gateway LAN software. Images were analyzed randomly by three of the authors without knowledge of clinicopathological data. For immunofluorescence, rabbit IgG anti-vimentin (Abcam, Cambridge, UK) and mouse anti-cytokeratin (Dako, Agilent Technologies, Glostrup, Denmark) were used at 1:500 dilution. Anti-rabbit IgG Alexa Fluor 488 and anti-mouse IgG Alexa Fluor 555 were used as secondary antibodies at 1:500 dilutions for 1 h. Immunofluorescence stainings were analyzed on a Zeiss AxioObserver Z1 microscope (Carl Zeiss, Oberkochen, Germany) with a Plan-Apochromat 40X/1.3 (NA = 1.3, working distance = 0.21 mm) or Plan-Apochromat 63X/1.4 (NA = 1.4, working distance = 0.19 mm) oil lens objective, a camera (AxioCam MRm; Carl Zeiss), and Apotome (ApoTome 2; Carl Zeiss) as previously described [26,36].

### 4.5. Western Blot

Protein extracts were obtained from SCC42B and SCC38 cells at 80–90% confluence. Equal amounts of proteins were fractionated on SDS–PAGE and transferred to PVDF membranes. Membranes were probed with mouse IgG anti-E-cadherin antibody (Becton Dickinson Transduction Laboratories, Erembodegem, Belgium) at 1:10,000 dilution, rabbit IgG anti-vimentin (Abcam, Cambridge, UK) at 1:1000 dilution, mouse anti-cytokeratin (Dako, Agilent Technologies, Denmark) at 1:500 dilution, mouse anti-FAK (Merck, Darmstadt, Germany) at 1:500 dilution, mouse anti-pY397 FAK (Becton Dickinson Transduction Laboratories, Erembodegem, Belgium) at 1:500 dilution, mouse anti-N-cadherin (Novus Biological, CO, USA) at 1:500 dilution, rabbit anti-ZEB1 (Novus Biological, CO, USA) at 1:500 dilution, or mouse anti-β-actin (Sigma Aldrich, St Louis, MO, USA) at 1:10,000 dilution. Bound antibodies were detected by using IRDye 800 CW or IRDye 700 IgG secondary antibodies. Odyssey Fc Imaging System was used for image acquisition and densitometric analysis.

### 4.6. Time-Lapse and Confocal Reflection Microscopy

Time-lapse microscopy imaging was performed on a Zeiss AxioObserver Z1 microscope (Carl Zeiss, Germany) with a Plan-Apochromat 40X/1.3 (NA = 1.3, working distance = 0.21 mm) or Plan-Apochromat 63X/1.4 (NA = 1.4, working distance = 0.19mm) oil lens objective, a camera (AxioCam MRm; Carl Zeiss), and Apotome (ApoTome 2; Carl Zeiss). z-stack images were taken with AxioVision module Z-stack (Zeiss). Three-dimensional reconstruction of z-stacks and two-dimensional projections were conducted using the Imaris 7.1 Software (Bitplane Scientific Software, Belfast, UK) and the Z Projection ImageJ plugin, respectively, 35. For confocal reflection microscopy, tumor-spheres were seeded on glass bottom 35mm-μ-dishes, incubated for different times and subsequently fixed with 4% formalin. Z-stack images were acquired using a Leica TCS-SP2 AOBS confocal microscopy with a HCX PL APO CS 40x/1.25 oil lens objective and a 488 nm Argon laser in reflection at room temperature.

### 4.7. Statistical Analysis

The two-tailed independent Student t-test was used to compare the variables between two groups. All data were derived from independent experiments. The level of statistical significance was set at 0.05 for all tests. TCGA head and neck cancer datasets were retrieved by using the UCSC Xena Functional Genomics Explorer [41]. To evaluate the association between the levels of each transcript and the clinical variables, the median value of transcript levels was used to dichotomize patients between high- or low-expressors. Clinical and molecular variables were compared between gene expression groups using χ2 or Fisher’s exact test for categorical variables. Statistical analyses for the impact of MMP-2 on overall survival was performed using Kaplan–Meier analysis (log-rank analysis for statistical significance). The median overall survival was 55 months (IC95%: 46–67). Multivariable Cox proportional hazards models were generated to assess statistical significance of prognostic factors with respect to OS. Besides MMP-2 expression, T, N, and EMT transcript levels were entered in the full multivariable models. Two-sided *p* values < 0.05 were considered significant. Analyses were performed using R software, version 3.5.1.

## 5. Conclusions

This work shows that the collective invasion of cancer cells is tuned by a balance between the expression levels of FAK and MMP-2 in cancer cells as well as on the presence of CAFs in the tumor microenvironment. Within the heterogeneous microenvironment of tumors, the highly invasive cancer cells that over-express MMP-2, and stromal fibroblasts, are capable of pulling the less motile cancer cells that lack functional FAK thus promoting collective migration of the whole cancer cell cluster. In head and neck SCCs, it is the high levels of MMP-2 gene expression, not of FAK, that associate with poor disease outcome. Thus, MMP-2 targeting should be re-considered as an effective strategy to halt cancer progression.

## Figures and Tables

**Table 1:** Correlations between *PTK2* and *MMP2* mRNA levels and clinical variables of the patients with head and neck squamous cell carcinomas included in the TCGA database.

		*PTK2* mRNA Levels			*MMP2* mRNA Levels		
	N	Low	High	*p*	Low	High	*p*
pT classification							
T1–T2	209	111	98	0.094	124	85	<0.0001
T3–T4	334	152	182		141	193	
pN classification							
N0	193	102	91	0.005	105	88	0.021
N+	347	159	188		159	188	
Distant metastasis							
M0	190	96	94	0.450	97	93	0.192
M+	66	37	29		39	27	
Disease stage							
I–II	119	67	52	0.098	70	49	0.007
III–IV	374	178	196		167	207	

## Supplementary Materials

The following are available online at https://www.mdpi.com/2072-6694/12/12/3708/s1, Figure S1: FRNK-induced silencing of active FAK in SCC42B cells, Figure S2: FAK and pFAK levels in SCC cell lines expressing FRNK, MMP-2, or FRNK + MMP-2 and in cells treated with siRNAs or PF-562271 inhibitor. Figure S3: FAK activity is required for efficient collective cell invasion of SCC40 cells. Figure S4: Quantification of the relative amount of MMP-2 in SCC cells transfected with empty vector or with FRNK-, MMP-2, or FRNK plus MMP-2 expressing vectors. Figure S5: Three-dimensional reconstructions of Z-stacked images of spheroids. Figure S6: High levels of MMP-2 protein in the invasive fronts of head and neck SCCs. Figure S7: Clinical outcome in patients with head and neck SCCs according to the expression levels of mesenchymal genes (TCGA dataset). Figure S8: Full-length blots of Westerns showed in Figure 6, Figure 7 and Figure 8. Table S1: Correlations of *PTK2*- and *MMP2*-mRNAs with expression levels of epithelial and mesenchymal genes in head and neck squamous cell carcinomas included in the TCGA database. Video S1: Representative video showing migration of a cell spheroid assembled with SCC42B cells and recorded for 5 h. Video S2: Representative video showing migration of a cell spheroid assembled with F-SCC42B cells and recorded for 5 h. Video S3: Representative video showing migration of a cell spheroid assembled with FM-SCC42B cells and recorded for 5 h.
