# Supplementary material for: Blockage of Squamous Cancer Cell Collective Invasion by FAK Inhibition Is Released by CAFs and MMP-2
Source: Cancers (Basel). 2020 Dec 10;12(12):3708. doi: 10.3390/cancers12123708 (PMC7764466; doi:10.3390/cancers12123708)
Supplement: Supplementary file 1 [file cancers-12-03708-s001.zip › cancers-1023595-suppl-final/cancers-1023595-suppl-final.docx]

Article

Blockage of Squamous Cancer Cell Collective Invasion by FAK Inhibition is Released by CAFs
and MMP-2

Inés Sáenz-de-Santa-María, Lucía Celada, Andrés San José Martínez, Tamara Cubiella and María-Dolores Chiara

Supplementary Materials:


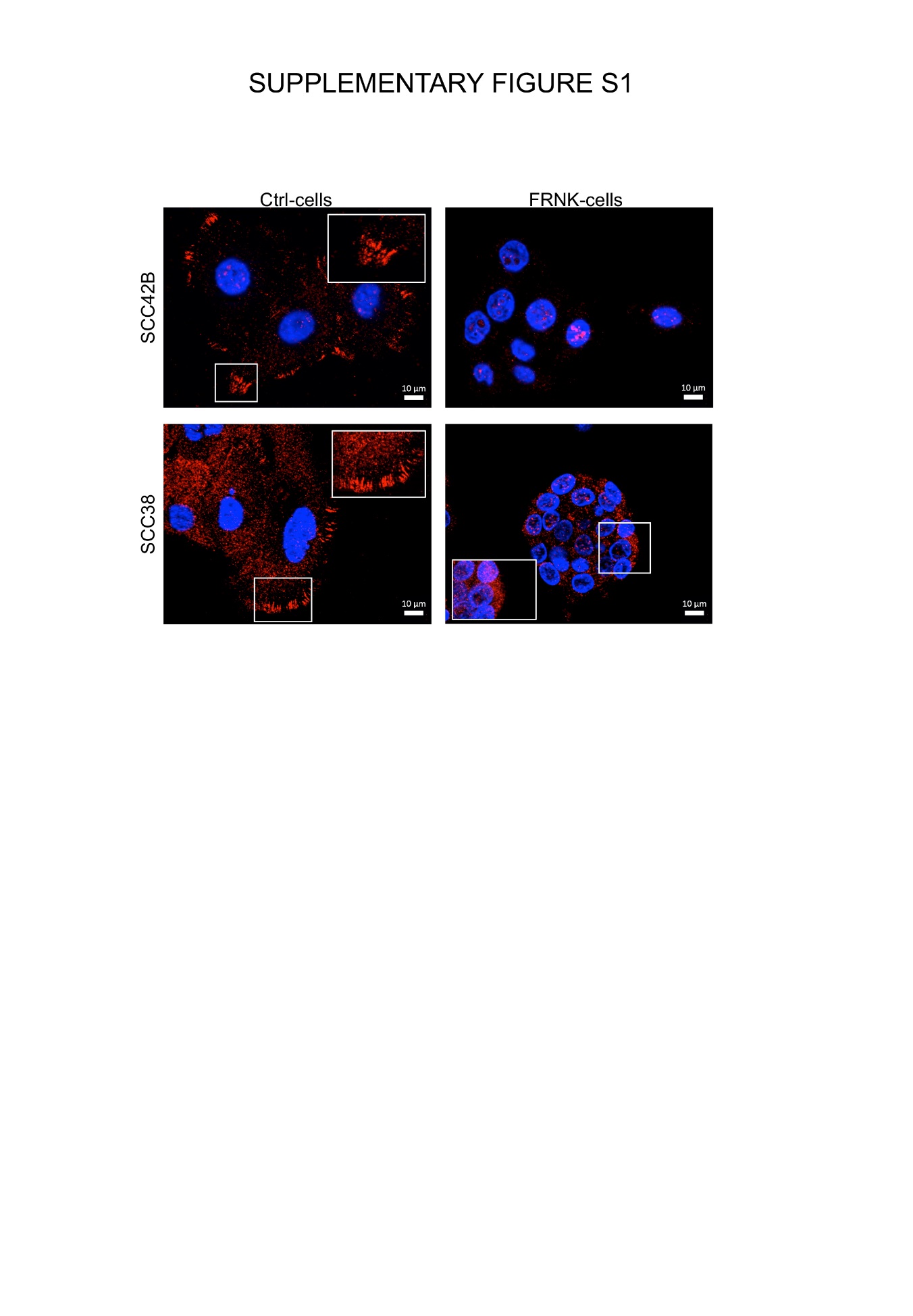


**Figure S1.** FRNK-induced silencing of active FAK in SCC42B cells.

Figure S2: FAK and pFAK levels in SCC cell lines expressing FRNK, MMP-2, or FRNK + MMP-2 and in cells treated with siRNAs or PF-562271 inhibitor
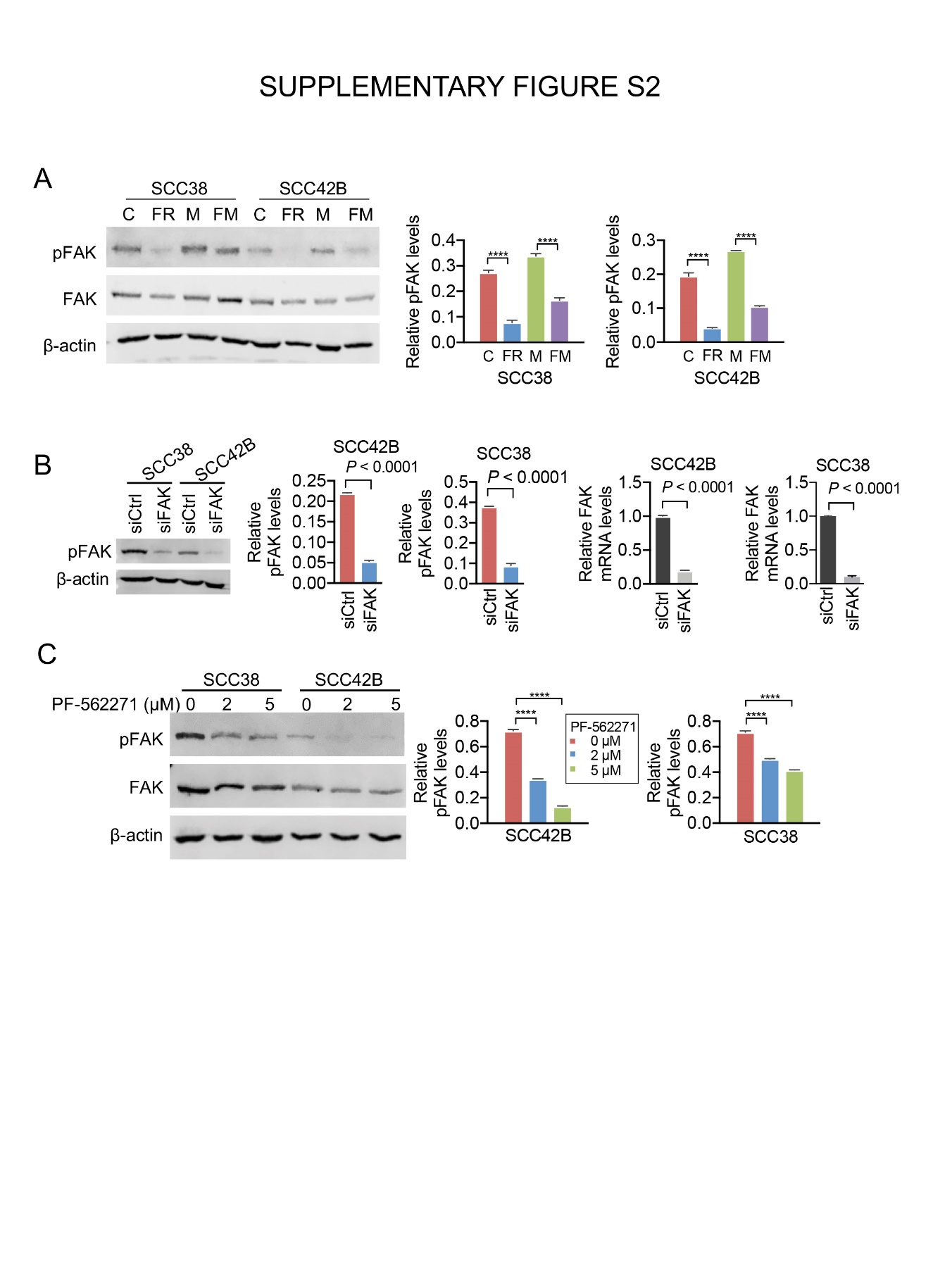


**Figure S2.** FAK and pFAK levels in SCC cell lines expressing FRNK, MMP-2, or FRNK + MMP-2 and in cells treated with siRNAs or PF-562271 inhibitor.


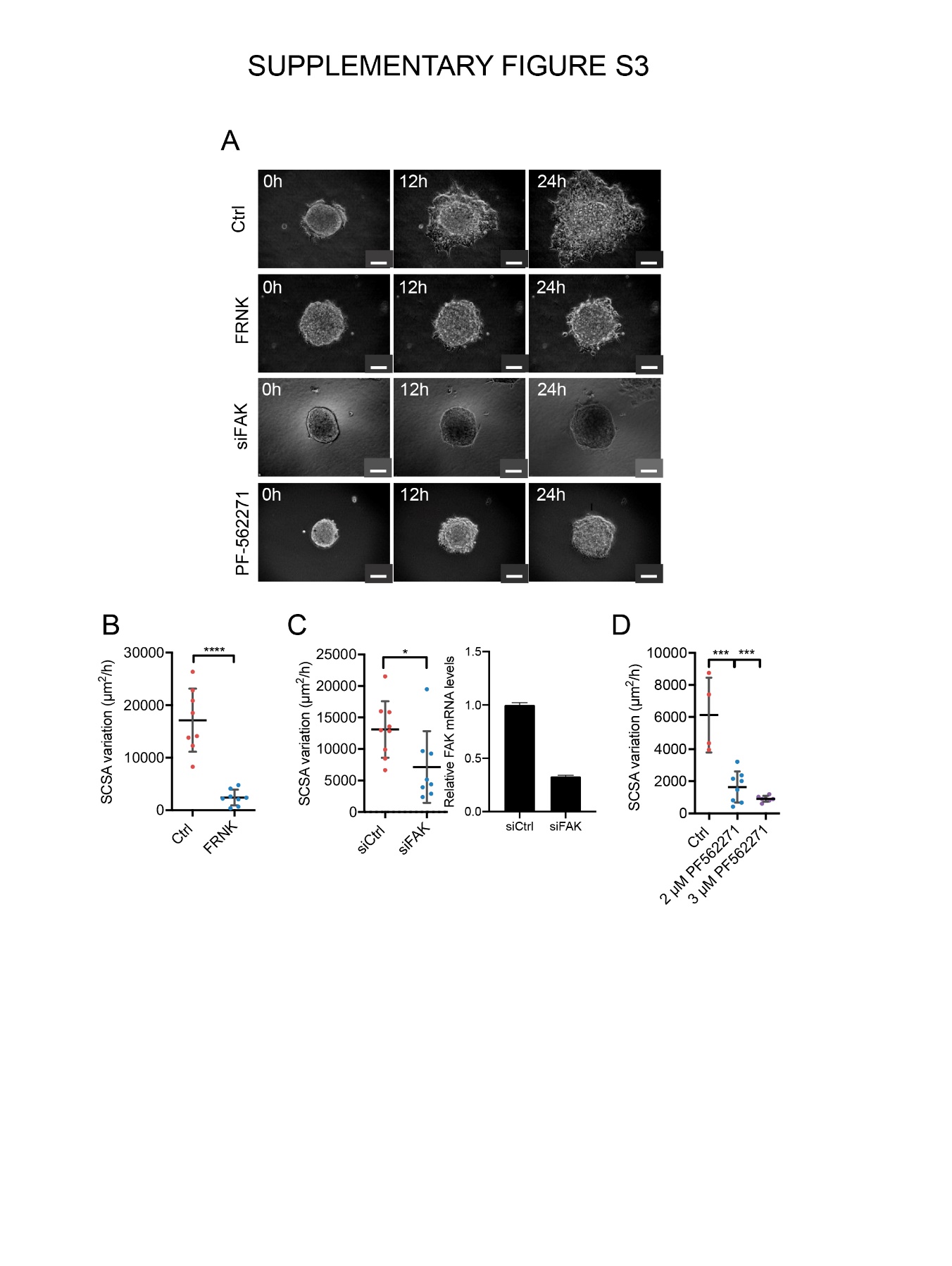


**Figure S3.** FAK activity is required for efficient collective cell invasion of SCC40 cells.


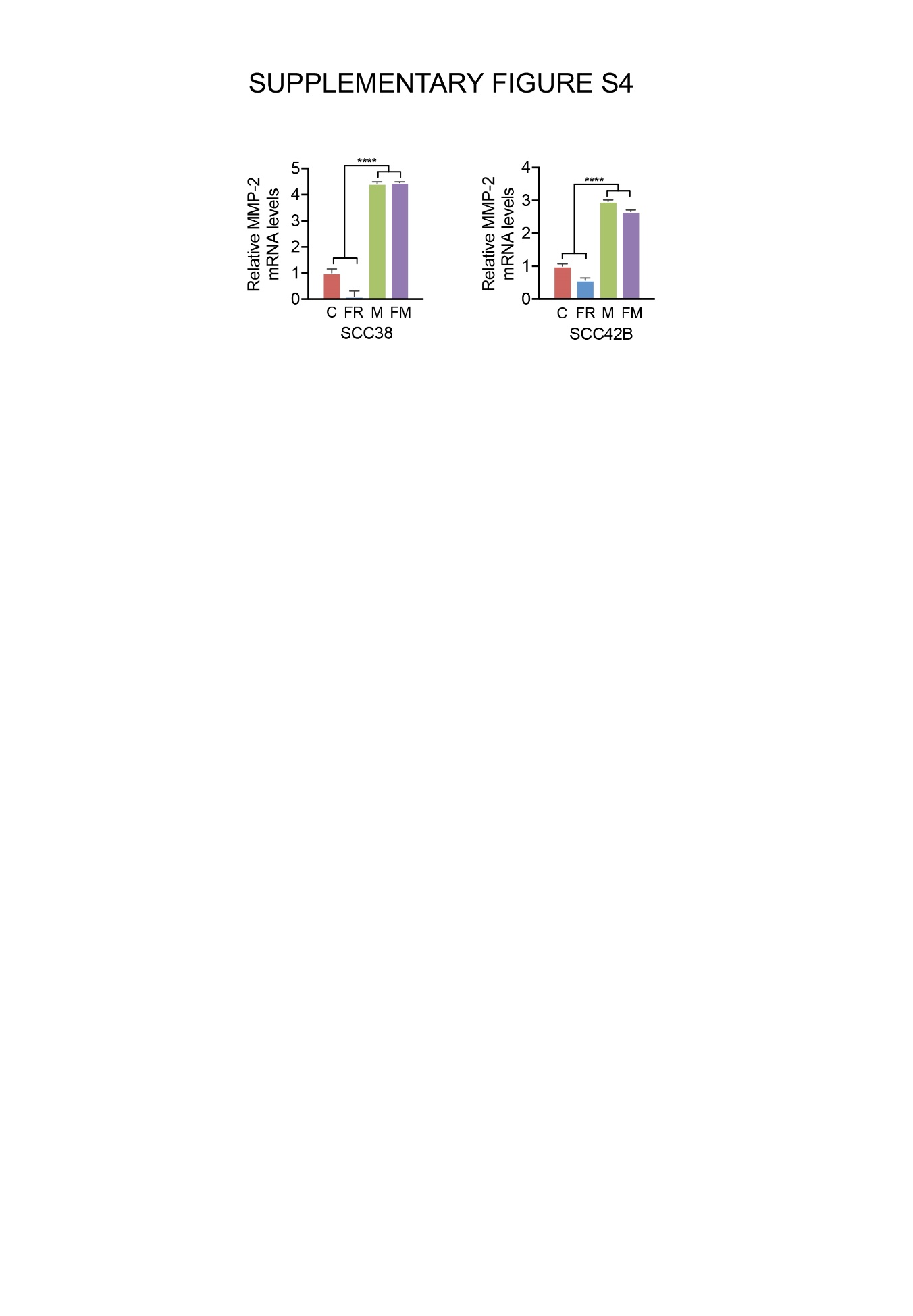


**Figure S4.** Quantification of the relative amount of MMP-2 in SCC cells transfected with empty vector or with FRNK-, MMP-2, or FRNK plus MMP-2 expressing vectors.


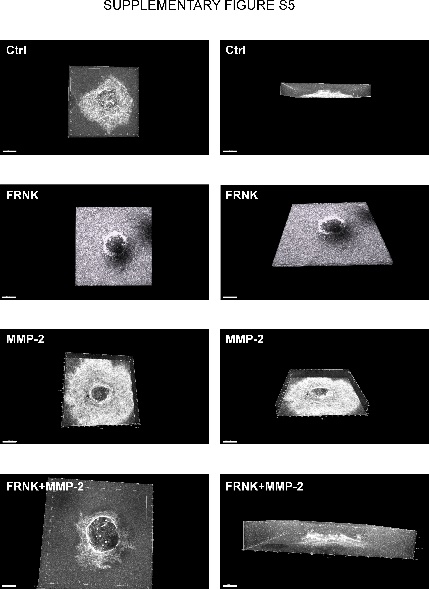


**Figure S5.** Three-dimensional reconstructions of Z-stacked images of spheroids.


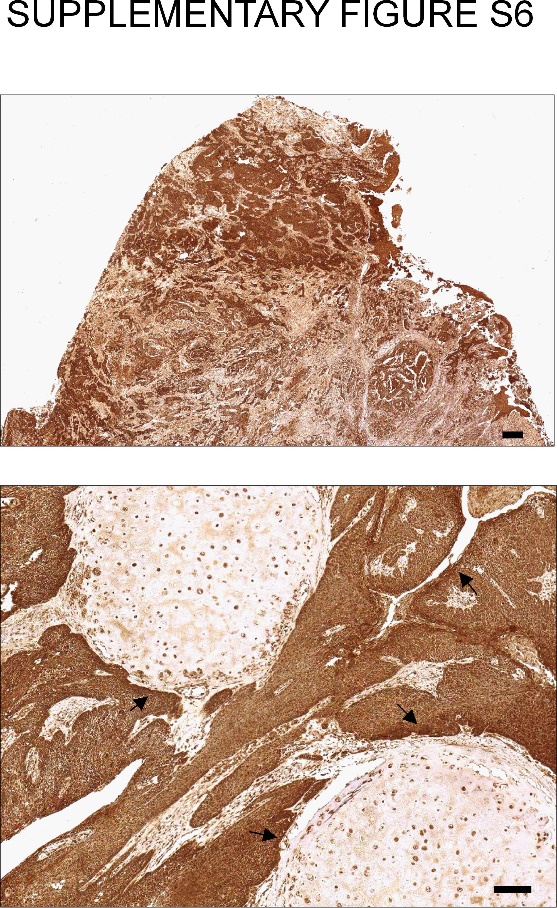


**Figure S6.** High levels of MMP-2 protein in the invasive fronts of head and neck SCCs.

**
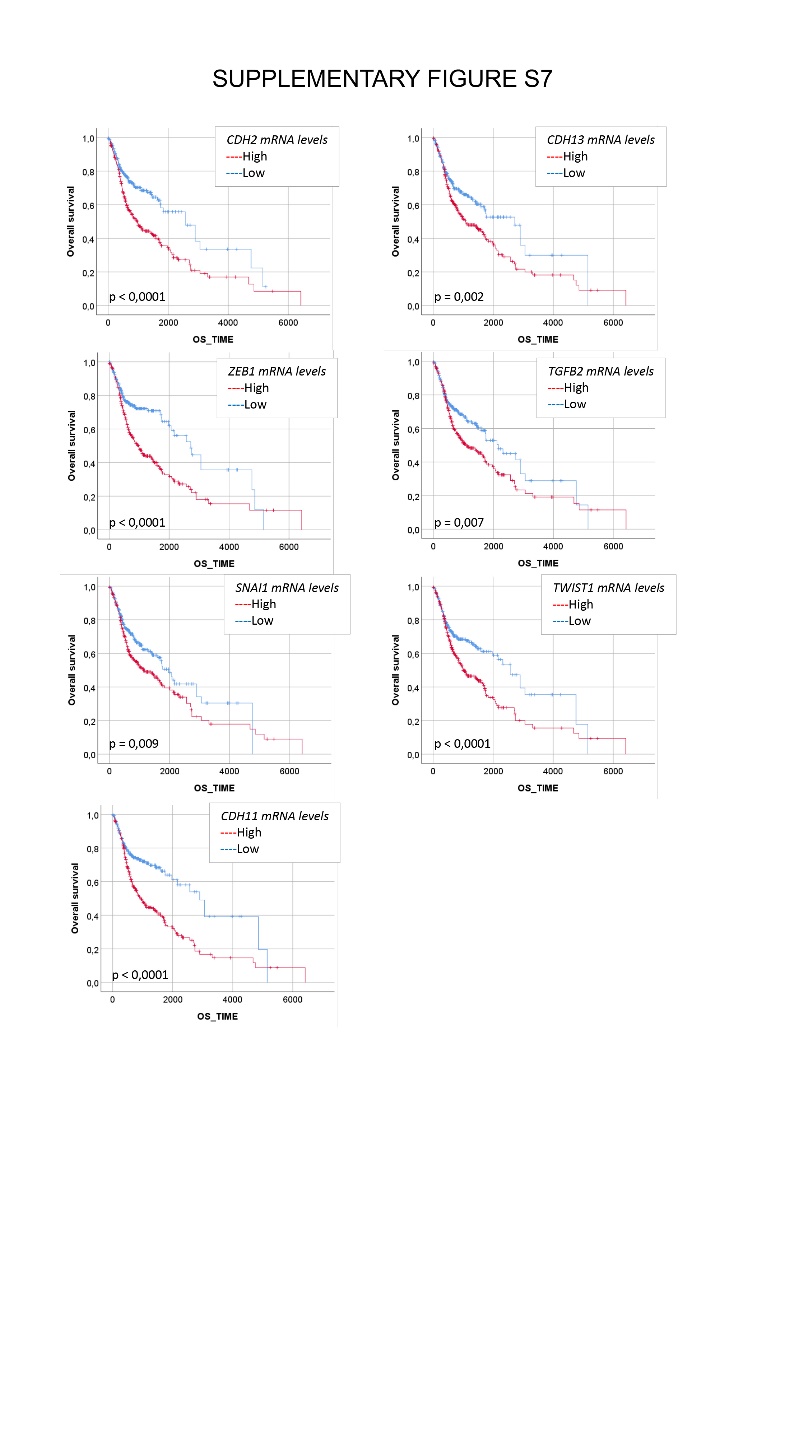
**

**Figure S7.** Clinical outcome in patients with head and neck SCCs according to the expression levels of mesenchymal genes (TCGA dataset).


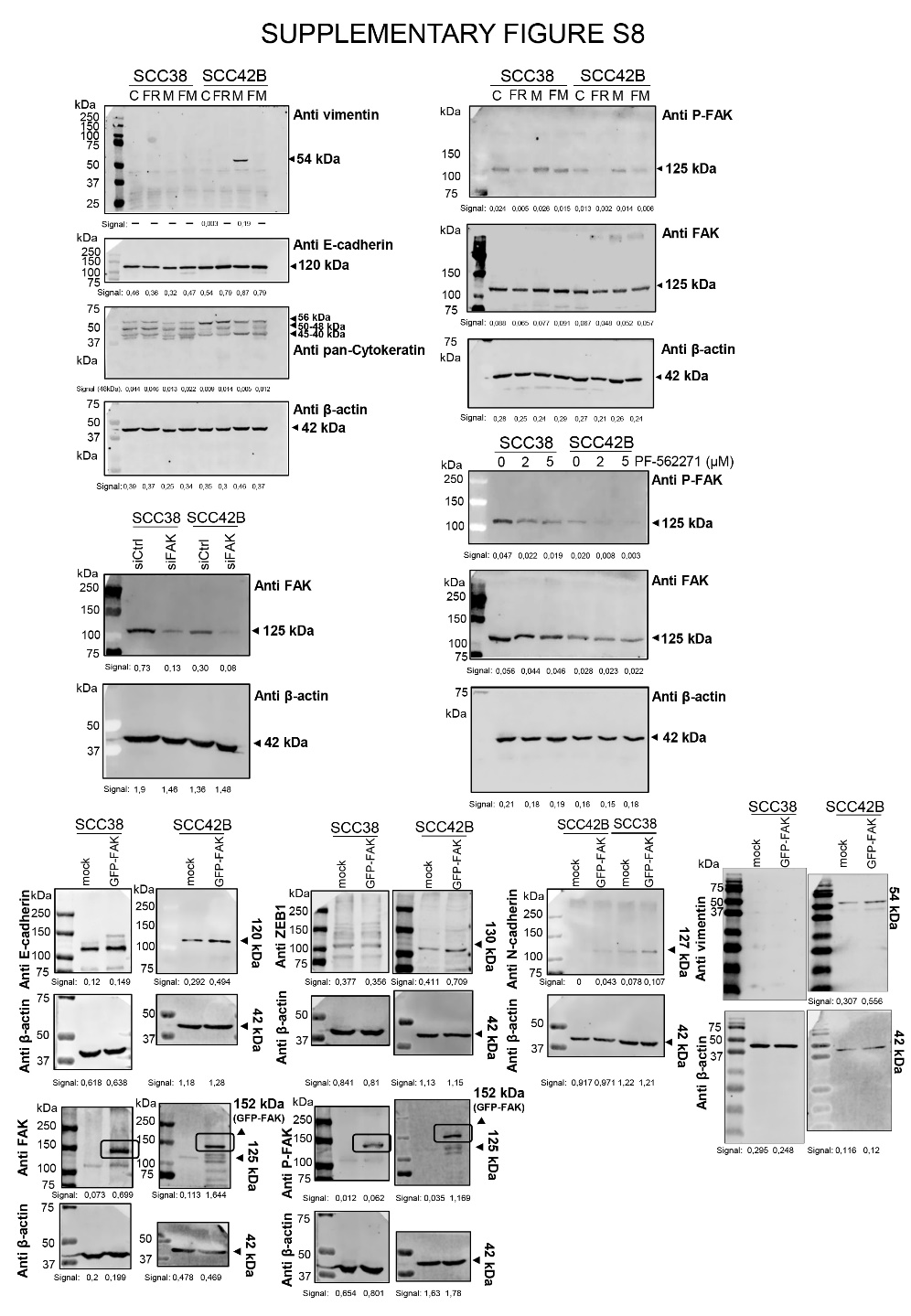


**Figure S8.** Full-length blots of Westerns showed in Figures 6–8. Table S1: Correlations of *PTK2*- and *MMP2*-mRNAs with expression levels of epithelial and mesenchymal genes in head and neck squamous cell carcinomas included in the TCGA database.

**Table S1.** Correlations of PTK2- and MMP2-mRNAs with expression levels of epithelial and mesenchymal genes in head and neck squamous cell carcinomas included in the TCGA database.

|  | | PTK2 | MMP2 | CDH1^+^ | CDH2^**^ | ZEB1^**^ | ZEB2^**^ | SNAI1^**^ | SNAI2^**^ | TWIST1^**^ | CDH11^**^ | CDH13^**^ | TGFB2^**^ | KRT13^+^ | KRT14^+^ | VIM^**^ | CLDN7^+^ | CLDN4^+^ | CDH26^+^ | EPCAM^+^ |
| --- | --- | --- | --- | --- | --- | --- | --- | --- | --- | --- | --- | --- | --- | --- | --- | --- | --- | --- | --- | --- |
| **PTK2** | Pearson correlation | 1 | ,237 | ,075 | ,213 | ,155 | ,140 | ,229 | ,406 | ,131 | ,339 | ,345 | ,298 | -,339 | -,014 | ,088 | -,246 | -,237 | -,180 | ,119 |
|  | Sig. (two-sided) |  | ,000 | ,075 | ,000 | ,000 | ,001 | ,000 | ,000 | ,002 | ,000 | ,000 | ,000 | ,000 | ,743 | ,036 | ,000 | ,000 | ,000 | ,005 |
|  | N | 566 | 566 | 566 | 566 | 566 | 566 | 566 | 566 | 566 | 566 | 566 | 566 | 566 | 566 | 566 | 566 | 566 | 566 | 566 |
| MMP2 | Pearson correlation | ,237 | 1 | -,077 | ,578 | ,664 | ,656 | ,624 | ,451 | ,669 | ,771 | ,477 | ,531 | -,271 | ,158 | ,651 | -,415 | -,311 | -,237 | -,123 |
|  | Sig. (two-sided) | ,000 |  | ,067 | ,000 | ,000 | ,000 | ,000 | ,000 | ,000 | ,000 | ,000 | ,000 | ,000 | ,000 | ,000 | ,000 | ,000 | ,000 | ,004 |
|  | N | 566 | 566 | 566 | 566 | 566 | 566 | 566 | 566 | 566 | 566 | 566 | 566 | 566 | 566 | 566 | 566 | 566 | 566 | 566 |
| CDH1 | Pearson correlation | ,075 | -,077 | 1 | -,200 | -,188 | -,194 | -,146 | -,039 | -,317 | -,099 | ,061 | -,063 | ,295 | ,348 | -,397 | ,266 | ,451 | ,332 | ,229 |
|  | Sig. (two-sided) | ,075 | ,067 |  | ,000 | ,000 | ,000 | ,000 | ,352 | ,000 | ,019 | ,144 | ,137 | ,000 | ,000 | ,000 | ,000 | ,000 | ,000 | ,000 |
|  | N | 566 | 566 | 566 | 566 | 566 | 566 | 566 | 566 | 566 | 566 | 566 | 566 | 566 | 566 | 566 | 566 | 566 | 566 | 566 |
| CDH2 | Pearson correlation | ,213 | ,578 | -,200 | 1 | ,559 | ,538 | ,439 | ,320 | ,587 | ,584 | ,356 | ,501 | -,211 | -,092 | ,599 | -,275 | -,334 | -,235 | -,080 |
|  | Sig. (two-sided) | ,000 | ,000 | ,000 |  | ,000 | ,000 | ,000 | ,000 | ,000 | ,000 | ,000 | ,000 | ,000 | ,029 | ,000 | ,000 | ,000 | ,000 | ,056 |
|  | N | 566 | 566 | 566 | 566 | 566 | 566 | 566 | 566 | 566 | 566 | 566 | 566 | 566 | 566 | 566 | 566 | 566 | 566 | 566 |
| ZEB1 | Pearson correlation | ,155 | ,664 | -,188 | ,559 | 1 | ,875 | ,526 | ,124 | ,482 | ,679 | ,356 | ,483 | -,110 | -,211 | ,659 | -,269 | -,243 | -,109 | -,141 |
|  | Sig. (two-sided) | ,000 | ,000 | ,000 | ,000 |  | ,000 | ,000 | ,003 | ,000 | ,000 | ,000 | ,000 | ,009 | ,000 | ,000 | ,000 | ,000 | ,009 | ,001 |
|  | N | 566 | 566 | 566 | 566 | 566 | 566 | 566 | 566 | 566 | 566 | 566 | 566 | 566 | 566 | 566 | 566 | 566 | 566 | 566 |
| ZEB2 | Pearson correlation | ,140 | ,656 | -,194 | ,538 | ,875 | 1 | ,559 | ,153 | ,513 | ,694 | ,272 | ,458 | -,155 | -,160 | ,754 | -,338 | -,289 | -,182 | -,218 |
|  | Sig. (two-sided) | ,001 | ,000 | ,000 | ,000 | ,000 |  | ,000 | ,000 | ,000 | ,000 | ,000 | ,000 | ,000 | ,000 | ,000 | ,000 | ,000 | ,000 | ,000 |
|  | N | 566 | 566 | 566 | 566 | 566 | 566 | 566 | 566 | 566 | 566 | 566 | 566 | 566 | 566 | 566 | 566 | 566 | 566 | 566 |
| SNAI1 | Pearson correlation | ,229 | ,624 | -,146 | ,439 | ,526 | ,559 | 1 | ,247 | ,551 | ,576 | ,320 | ,458 | -,301 | -,003 | ,546 | -,271 | -,186 | -,285 | -,044 |
|  | Sig. (two-sided) | ,000 | ,000 | ,000 | ,000 | ,000 | ,000 |  | ,000 | ,000 | ,000 | ,000 | ,000 | ,000 | ,936 | ,000 | ,000 | ,000 | ,000 | ,292 |
|  | N | 566 | 566 | 566 | 566 | 566 | 566 | 566 | 566 | 566 | 566 | 566 | 566 | 566 | 566 | 566 | 566 | 566 | 566 | 566 |
| SNAI2 | Pearson correlation | ,406 | ,451 | -,039 | ,320 | ,124 | ,153 | ,247 | 1 | ,329 | ,407 | ,499 | ,304 | -,464 | ,476 | ,268 | -,578 | -,523 | -,366 | -,215 |
|  | Sig. (two-sided) | ,000 | ,000 | ,352 | ,000 | ,003 | ,000 | ,000 |  | ,000 | ,000 | ,000 | ,000 | ,000 | ,000 | ,000 | ,000 | ,000 | ,000 | ,000 |
|  | N | 566 | 566 | 566 | 566 | 566 | 566 | 566 | 566 | 566 | 566 | 566 | 566 | 566 | 566 | 566 | 566 | 566 | 566 | 566 |
| TWIST1 | Pearson correlation | ,131 | ,669 | -,317 | ,587 | ,482 | ,513 | ,551 | ,329 | 1 | ,582 | ,283 | ,419 | -,325 | ,008 | ,627 | -,370 | -,322 | -,309 | -,124 |
|  | Sig. (two-sided) | ,002 | ,000 | ,000 | ,000 | ,000 | ,000 | ,000 | ,000 |  | ,000 | ,000 | ,000 | ,000 | ,846 | ,000 | ,000 | ,000 | ,000 | ,003 |
|  | N | 566 | 566 | 566 | 566 | 566 | 566 | 566 | 566 | 566 | 566 | 566 | 566 | 566 | 566 | 566 | 566 | 566 | 566 | 566 |
| CDH11 | Pearson correlation | ,339 | ,771 | -,099 | ,584 | ,679 | ,694 | ,576 | ,407 | ,582 | 1 | ,396 | ,473 | -,268 | -,072 | ,629 | -,359 | -,319 | -,174 | ,049 |
|  | Sig. (two-sided) | ,000 | ,000 | ,019 | ,000 | ,000 | ,000 | ,000 | ,000 | ,000 |  | ,000 | ,000 | ,000 | ,088 | ,000 | ,000 | ,000 | ,000 | ,248 |
|  | N | 566 | 566 | 566 | 566 | 566 | 566 | 566 | 566 | 566 | 566 | 566 | 566 | 566 | 566 | 566 | 566 | 566 | 566 | 566 |
| CDH13 | Pearson correlation | ,345 | ,477 | ,061 | ,356 | ,356 | ,272 | ,320 | ,499 | ,283 | ,396 | 1 | ,396 | -,290 | ,326 | ,302 | -,429 | -,326 | -,247 | -,212 |
|  | Sig. (two-sided) | ,000 | ,000 | ,144 | ,000 | ,000 | ,000 | ,000 | ,000 | ,000 | ,000 |  | ,000 | ,000 | ,000 | ,000 | ,000 | ,000 | ,000 | ,000 |
|  | N | 566 | 566 | 566 | 566 | 566 | 566 | 566 | 566 | 566 | 566 | 566 | 566 | 566 | 566 | 566 | 566 | 566 | 566 | 566 |
| TGFB2 | Pearson correlation | ,298 | ,531 | -,063 | ,501 | ,483 | ,458 | ,458 | ,304 | ,419 | ,473 | ,396 | 1 | -,249 | ,028 | ,493 | -,236 | -,226 | -,250 | -,046 |
|  | Sig. (two-sided) | ,000 | ,000 | ,137 | ,000 | ,000 | ,000 | ,000 | ,000 | ,000 | ,000 | ,000 |  | ,000 | ,499 | ,000 | ,000 | ,000 | ,000 | ,272 |
|  | N | 566 | 566 | 566 | 566 | 566 | 566 | 566 | 566 | 566 | 566 | 566 | 566 | 566 | 566 | 566 | 566 | 566 | 566 | 566 |
| KRT13 | Pearson correlation | -,339 | -,271 | ,295 | -,211 | -,110 | -,155 | -,301 | -,464 | -,325 | -,268 | -,290 | -,249 | 1 | -,056 | -,277 | ,543 | ,591 | ,539 | ,034 |
|  | Sig. (two-sided) | ,000 | ,000 | ,000 | ,000 | ,009 | ,000 | ,000 | ,000 | ,000 | ,000 | ,000 | ,000 |  | ,184 | ,000 | ,000 | ,000 | ,000 | ,415 |
|  | N | 566 | 566 | 566 | 566 | 566 | 566 | 566 | 566 | 566 | 566 | 566 | 566 | 566 | 566 | 566 | 566 | 566 | 566 | 566 |
| KRT14 | Pearson correlation | -,014 | ,158 | ,348 | -,092 | -,211 | -,160 | -,003 | ,476 | ,008 | -,072 | ,326 | ,028 | -,056 | 1 | -,094 | -,289 | -,104 | -,175 | -,337 |
|  | Sig. (two-sided) | ,743 | ,000 | ,000 | ,029 | ,000 | ,000 | ,936 | ,000 | ,846 | ,088 | ,000 | ,499 | ,184 |  | ,026 | ,000 | ,013 | ,000 | ,000 |
|  | N | 566 | 566 | 566 | 566 | 566 | 566 | 566 | 566 | 566 | 566 | 566 | 566 | 566 | 566 | 566 | 566 | 566 | 566 | 566 |
| VIM | Pearson correlation | ,088 | ,651 | -,397 | ,599 | ,659 | ,754 | ,546 | ,268 | ,627 | ,629 | ,302 | ,493 | -,277 | -,094 | 1 | -,416 | -,396 | -,341 | -,228 |
|  | Sig. (two-sided) | ,036 | ,000 | ,000 | ,000 | ,000 | ,000 | ,000 | ,000 | ,000 | ,000 | ,000 | ,000 | ,000 | ,026 |  | ,000 | ,000 | ,000 | ,000 |
|  | N | 566 | 566 | 566 | 566 | 566 | 566 | 566 | 566 | 566 | 566 | 566 | 566 | 566 | 566 | 566 | 566 | 566 | 566 | 566 |
| CLDN7 | Pearson correlation | -,246 | -,415 | ,266 | -,275 | -,269 | -,338 | -,271 | -,578 | -,370 | -,359 | -,429 | -,236 | ,543 | -,289 | -,416 | 1 | ,662 | ,503 | ,408 |
|  | Sig. (two-sided) | ,000 | ,000 | ,000 | ,000 | ,000 | ,000 | ,000 | ,000 | ,000 | ,000 | ,000 | ,000 | ,000 | ,000 | ,000 |  | ,000 | ,000 | ,000 |
|  | N | 566 | 566 | 566 | 566 | 566 | 566 | 566 | 566 | 566 | 566 | 566 | 566 | 566 | 566 | 566 | 566 | 566 | 566 | 566 |
| CLDN4 | Pearson correlation | -,237 | -,311 | ,451 | -,334 | -,243 | -,289 | -,186 | -,523 | -,322 | -,319 | -,326 | -,226 | ,591 | -,104 | -,396 | ,662 | 1 | ,548 | ,250 |
|  | Sig. (two-sided) | ,000 | ,000 | ,000 | ,000 | ,000 | ,000 | ,000 | ,000 | ,000 | ,000 | ,000 | ,000 | ,000 | ,013 | ,000 | ,000 |  | ,000 | ,000 |
|  | N | 566 | 566 | 566 | 566 | 566 | 566 | 566 | 566 | 566 | 566 | 566 | 566 | 566 | 566 | 566 | 566 | 566 | 566 | 566 |
| CDH26 | Pearson correlation | -,180 | -,237 | ,332 | -,235 | -,109 | -,182 | -,285 | -,366 | -,309 | -,174 | -,247 | -,250 | ,539 | -,175 | -,341 | ,503 | ,548 | 1 | ,241 |
|  | Sig. (two-sided) | ,000 | ,000 | ,000 | ,000 | ,009 | ,000 | ,000 | ,000 | ,000 | ,000 | ,000 | ,000 | ,000 | ,000 | ,000 | ,000 | ,000 |  | ,000 |
|  | N | 566 | 566 | 566 | 566 | 566 | 566 | 566 | 566 | 566 | 566 | 566 | 566 | 566 | 566 | 566 | 566 | 566 | 566 | 566 |
| EPCAM | Pearson correlation | ,119 | -,123 | ,229 | -,080 | -,141 | -,218 | -,044 | -,215 | -,124 | ,049 | -,212 | -,046 | ,034 | -,337 | -,228 | ,408 | ,250 | ,241 | 1 |
|  | Sig. (two-sided) | ,005 | ,004 | ,000 | ,056 | ,001 | ,000 | ,292 | ,000 | ,003 | ,248 | ,000 | ,272 | ,415 | ,000 | ,000 | ,000 | ,000 | ,000 |  |
|  | N | 566 | 566 | 566 | 566 | 566 | 566 | 566 | 566 | 566 | 566 | 566 | 566 | 566 | 566 | 566 | 566 | 566 | 566 | 566 |

^*^Epithelial genes; ^**^Mesenchymal genes.

| 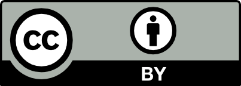 | © 2020 by the authors. Submitted for possible open access publication under the terms and conditions of the Creative Commons Attribution (CC BY) license (http://creativecommons.org/licenses/by/4.0/). |
| --- | --- |
